# Fruit juice – a scoping review for Nordic Nutrition Recommendations 2023

**DOI:** 10.29219/fnr.v68.10463

**Published:** 2024-02-06

**Authors:** Magdalena Rosell, Christine Delisle Nyström

**Affiliations:** Department of Biosciences and Nutrition, Karolinska Institutet, Stockholm, Sweden

**Keywords:** fruit juice, fruit, beverages, dietary guidelines

## Abstract

Fruit juice has a similar nutrient content as whole fruit and may in this respect be part of a healthy diet. However, a lower amount or lack of fibre and a higher concentration of sugars and energy could also contribute to less satiation and increase the risk of excess energy intake. The aim of this scoping review is to describe the overall evidence for the role of fruit juice as a basis for setting and updating food-based dietary guidelines in the Nordic Nutrition Recommendations 2023. PubMed was searched for systematic reviews and meta-analyses and evidence was extracted on relevant health outcomes. Current available evidence indicates that low to moderate consumption of fruit juice is not associated with an apparent risk of chronic diseases and may have protective effects on cardiovascular disease. The association between the intake of fruit juice and weight gain remains unclear and might be of concern for some groups of people. Overall, the evidence regarding health effects of fruit juice is limited.

## Popular scientific summary

- Except for fibre, fruit juice in general has a similar nutritional value as whole fruit
- Research on health effects of fruit juice is limited
- Recent evidence suggests that low to moderate consumption of fruit juice is not associated with risk of chronic disease
- The association between intake of fruit juice and body weight and dental health is unclear

Fruit juice can be defined as the liquid obtained from the edible part of fruit, which is ripe and fresh or preserved by chilling or freezing (1). Fruits can be culinary defined as the edible flesh that surrounds the seeds produced by a tree or other plant, that has a sweet or tart taste (2), while berries can be defined as small, fleshy fruits (3). Vegetable juice, including juice from foods botanically defined as fruits, such as tomatoes, but culinary defined as vegetables, is not addressed in this scoping review. Fruit juice can be obtained from a single fruit, such as orange juice, apple juice, pineapple juice, and pomegranate juice, or be a mixture of several fruits. The EU legislation Directive 2012/12/EU, which defines the term juice in the Nordic and Baltic countries, requires that fruit juice must be directly derived from the constituent fruit, with a similar composition to fruits they come from, including all elements naturally present in fruit. Components such as preservatives, sugars, sweeteners, and colourants cannot be added or removed (1). Hence, flavour, pulp, and cells that are separated from the juice during the process may be restored to the juice. Fruit juice may also be produced from concentrated fruit juice by adding the same amount of water as extracted during the concentration (1). This also corresponds to the definition of 100% fruit juice, while other terms should be used for beverages that contain less than 100% fruit juice (4). In nutrition research, the term fruit drink is often used for a beverage other than fruit juice. This scoping review is focused on health aspects of fruit juice in the sense of 100% fruit juice with no added sugar; however, the term fruit juice is not always clearly defined in studies investigating peoples’ habitual dietary habits.

In general, fruit juice has a similar nutritional value as whole fruit, except for fibre (4, 5). Vitamin C may also be lower, but may be added to the juice (4, 5). The content of phytochemicals may even be higher in commercial juice compared with home-squeezed juice since these compounds may be transferred from peels and seeds into the juice in the process (4). However, the bioavailability of these compounds may vary (4). The content of nutrients and phytochemicals may contribute to positive health effects as also seen for whole fruit. On the other hand, a lower amount or lack of fibre could also contribute to less satiation and increase the risk of excess energy intake (4). This scoping review will summarise the evidence for the role of fruit juice for health-related outcomes as a basis for setting and updating food-based dietary guidelines in the Nordic Nutrition Recommendations 2023 (Box 1). The search strategy was focused on systematic reviews and meta-analyses of observational and intervention studies summarising epidemiologic evidence in this area.

Box 1 Background papers for Nordic Nutrition Recommendations 2023

- This paper is one of many scoping reviews commissioned as part of the Nordic Nutrition Recommendations 2023 (NNR2023) project (6)
- The papers are included in the extended NNR2023 report but, for transparency, these scoping reviews are also published in Food & Nutrition Research
- The scoping reviews have been peer reviewed by independent experts in the research field according to the standard procedures of the journal
- The scoping reviews have also been subjected to public consultations (see report to be published by the NNR2023 project)
- The NNR2023 committee has served as the editorial board
- While these papers are a main fundament, the NNR2023 committee has the sole responsibility for setting dietary reference values in the NNR2023 project

## Methods

This scoping review is based on the same methodology and search strategy described previously (7). Systematic reviews and meta-analyses for fruit juice were selected in the same literature search. No qualified or *de novo* NNR2023 systematic review were used for the current review (8). Of the 166 articles identified in the main search, 30 articles investigated fruit juice. Three of these articles included cancer as an outcome, but since cancer was not specifically searched for in the main search, an additional search regarding cancer as an outcome was done using the following search string:

“fruit juice”[Title/Abstract] AND cancer[Title/Abstract] AND 2011:2022 [pdat] AND (meta-analysis[Filter] OR systematic review[Filter])

This search generated seven articles, of which two additional articles were identified regarding cancer, resulting in 32 articles in total (**Supplementary material**). For the summary below, articles were selected based on the most recent reviews, comprehensiveness and quality checked by using the adjusted AMSTAR 2-NNR (6, 9). Systematic reviews and meta-analyses of prospective studies and randomised controlled trials were of primary interest, but cross-sectional studies were also considered if no other evidence was available. For the section on mechanisms, a general search approach was also used.

## Diet intake in Nordic and Baltic countries

According to data from national dietary surveys in adult men and women, the mean intake of juice (the type of juice is not specified) ranges from 35 to 114 g/day among the different Nordic and Baltic countries, with large individual variations within the countries (no data was available for Lithuania) (10). The highest intakes are seen in Norway and Iceland and the lowest in Estonia and Latvia. In all countries, the intake of juice was higher in men than in women (10).

## Health outcomes relevant for Nordic and Baltic countries

Some of the included studies below utilize the term ‘fruit juice’, while others use the term ‘100% fruit juice’. Furthermore, in some of the studies, other types of juice, such as fruit drinks, have been investigated. The terms used in the summary below are the same terms that are used in the different studies. The results below are based on prospective studies unless otherwise indicated.

Recent meta-analyses of prospective studies have reported no associations (11–13) or indications of inverse associations (11) between the intake of fruit juice and the risk of cardiovascular disease incidence and/or mortality, comparing high versus low intake. A beneficial effect was noted particularly for stroke. One meta-analysis reported an increased risk of cardiovascular disease mortality; however, this was based only on one study (14). J-shaped associations have been demonstrated for 100% fruit juice, with the largest risk reductions seen at around 80 ml/d for cardiovascular disease and stroke (15). However, all estimates are based on few studies and the quality of evidence was considered very low (11, 14), low (11), and moderate (15), respectively, using the GRADE criteria. Regarding cancer, one meta-analysis reported that a high intake of fruit juice was associated with a small increased risk (6%) of overall cancer compared with low intake, which was also supported by a linear dose-response association (16); however, the evidence was considered poor due to indication of publication bias (16). Slightly increased risks (3–4%) have also been reported for breast cancer (17) and prostate cancer (18) when comparing high versus low intake, but the latter was rated as low confidence according to AMSTAR 2-NNR. No association has been reported for all-cause mortality (12, 14). For type 2 diabetes, no association was seen for 100% fruit juice in the most recent and comprehensive review, whereas a small increased risk was reported for fruit juice unspecified and a distinct increased risk for fruit drinks (19). Using the World Cancer Research Fund (WCRF) criteria, the evidence was considered limited – no conclusion for 100% fruit juice and limited – suggestive for fruit juice (unspecified) and fruit drinks (19).

A few systematic reviews and meta-analyses were also found for other outcomes. An adverse association between the intake of fruit juice and incident gout was reported, based on two studies that did not differentiate between pure fruit juice (the term used by the authors) and fruit drinks; the certainty of evidence was considered very low using the GRADE criteria (20). Regarding dental health, a recent systematic review on prospective studies showed no association between the intake of 100% fruit juice and tooth erosion or dental caries in children and adolescents (21). The same review also included trials, with data only available for adults, which suggested possible associations between the intake of 100% fruit juice and tooth erosion and markers of dental caries (21). However, the consumption of fruit juice (frequency and/or amounts) in these trials was often higher compared with average intakes (21). The trials also involved other methodological concerns, such as small number of participants, different methodologies for assessing the outcomes, and possible bias arising from the randomization processes (21).

Meta-analyses on intermediate risk factors have indicated U-shaped associations between the intake of 100% fruit juice and risk of hypertension (22) and metabolic syndrome (23); the certainty of evidence was considered low and moderate, respectively, using the GRADE criteria (22, 23). Beneficial effects on blood pressure, arterial compliance, and endothelial function are also suggested by short-term trials, while no effects were seen on blood lipids in the latest meta-analysis (15). Beneficial effects on blood uric acid (24) and antioxidant status (25, 26) have been indicated. However, the reports on antioxidant status were rated as low confidence according to AMSTAR 2‑NNR, and updated meta-analyses are warranted.

Regarding body weight, a pooled analysis from 2013 based on three cohorts in the USA indicated that the intake of fruit juice was associated with an increased risk of weight gain, more noticeable in people with overweight or obesity (27, 28). Updated meta-analyses are warranted to further elucidate this possible association. No effects on body weight outcomes were reported in a recent meta-analysis of short-term trials (15). In children, the intake of 100% fruit juice was associated with a small risk of weight gain in younger children (under the age of five or seven) (29, 30), of no clinical significance at the individual level and unclear significance at the population level (29). No clear association was found for older children in these two reports.

## Mechanisms

With the exception of fibre, fruit juice may contain similar amounts of nutrients as whole fruits (5) and a vast range of phytochemicals, such as carotenoids and polyphenols (4), which could mediate similar health effects as whole fruit (7). Possible mechanisms in relation to cardiovascular disease include antioxidant effects, improved endothelial function, decreased platelet aggregation, anti-inflammatory effects, and preventing hyperhomocysteinaemia (31). However, similar to sugar-sweetened beverages, which are associated with an increased risk of obesity and cardiometabolic diseases (32), fruit juice also has a low amount or lack of fibre and a sugar and energy content similar to sugar-sweetened beverages, which raises the concern of excess energy intake. An excessive intake of fructose may also affect the immune homeostasis and enhance tumour growth (33).

## Food-based dietary guidelines

Current available evidence indicates that low to moderate consumption of fruit juice is not associated with an apparent risk of chronic diseases. Beneficial effects on cardiovascular disease are indicated, while possible adverse effects on body weight and tooth erosion remain to be further elucidated. Overall, the evidence regarding health effects of fruit juice is very limited, and qSRs are lacking.

With exception of fibre, fruit juice in general has a similar nutritional value as the whole fruit, and a low to moderate intake of fruit juice may in this respect be part of a healthy diet. Dietary patterns associated with fruit juice are also relevant to consider, as studies have shown that the consumption of fruit juice is linked to better diet quality in general (34–36). A possible increased risk of excess energy intake might be of particular concern in people with overweight and obesity and in young children. For example, for a young child, a small glass of juice corresponds to a higher percentage of their daily energy intake compared with older children and adults, which gives reason to be cautious with excessive consumption. To promote eating behaviours associated with eating whole fruits, the American Academy of Paediatrics (AAP) does not recommend fruit juice intake before age 12 months and limited amounts during the second year (37). Avoiding drinking fruit juice between meals may also be relevant to prevent possible tooth erosion due to fruit juice consumption (38).

The studies on fruit juice highlight the importance of analysing non-linear dose-response associations in nutrition research (39). Gaps for future research include further investigation of health effects of fruit juice in both prospective observational studies and trials and in different age groups, taking amounts and dose-response associations into account. This would also include comparisons of health effects from fruit juice and whole fruit to further elucidate the differences and underlying mechanisms.

The point of departure of this scoping review is the accompanying scoping review conducted for vegetables, fruits, and berries, and the current review has similar limitations described previously (7). The search strategy was focused on systematic reviews and meta-analyses only, and original studies might have added further information. The search strategy also focused on fruit juice in general, and not on specific types of fruit juice, such as orange juice or apple juice. Systematic reviews and meta-analyses on different types of juices and their components might provide additional information on possible health effects and underlying mechanisms.
