# Supplementary material for: Fruit juice – a scoping review for Nordic Nutrition Recommendations 2023
Source: Food Nutr Res. 2024 Feb 6;68:10.29219/fnr.v68.10463. doi: 10.29219/fnr.v68.10463 (PMC10870970; doi:10.29219/fnr.v68.10463)
Supplement: Supplementary file 1 [file FNR-68-10463-s001.pdf]

Overview of the articles

The articles listed below were identified in the literature search. The articles marked in bold are selected for the summary in the chapter.

Extracted data and comments for all articles, as well as the quality of the selected articles, have been checked by Lars T. Fadnes, except for the studies on fruit juice and potatoes. The extracted data for the selected articles on fruit juice and potatoes have been checked by Christine Delisle Nyström.

The main search was done 4th of April 2021. A preliminary update of the search was done in November 2021 and again in May 2022.
A full update of the search was done 1st of February 2023, with a complementation 28th of February 2023 (since the search string in the first update was only until 2022).

Umbrella reviews for NCDs (some more umbrella reviews are listed further down, for asthma and fruit juice)

**Author year**	**Scope of the umbrella review**	**Exposures**	**Outcomes**	**Results**	**Quality/comments**
Wang et al 2022 (1) *Added in updated search 3*	22 Nov 2021<br>n=24 (SR or MA)	Diet, nutrition	Biliary tract diseases (cancer, gallstone)	Fruits and vegetable seem protective	Not specifically investigating fruits and vegetables
Webster et al 2021 (2)<br>*Added in updated search 3*	Nov 2020<br>Meta-analyses<br>n=16	Diet (including food groups, dietary patterns)	Hip fracture	Inverse associations for fruits and vegetables combined – low quality of evidence.	The latest meta-analysis by Brondani 2019 is included below.
Wallace et al 2019 (3)	May 2019<br>n=not clearly described, but a list presented in the table	Fruits and vegetables separately and combined	Incidence and/or mortality of NCDs		Not identified in the search (not classified as a systematic review).<br>No clear description of the selection of articles.<br>Mix of narrative approach
Kwok et al 2019 (4)	Aug 2018	Several individual dietary components	Risk of CVD or mortality		This article is covered by Aune.
Yip et al 2019 (5)	April 2018<br>Meta-analyses<br>n=64	Fruits and vegetables separately and combined.<br>dose-response	Incidence and/or mortality of NCDs (Burden of disease)	Summary of relevant meta-analysis	Seems good. The included studied are checked. There are also additional studies after April 2018.
Angelino et al 2019 (6)	Jan 2017<br>Reviews of cohort studies or RCTs<br>n=58 (fruit)<br>n=59 (vegetables)	Fruits and vegetables separately (not combined)	All types of NCDs (no intermediary biomarkers)	Fruit<br>Probable: CVC<br>Possible: depression, pancreatic disease<br>Limited: Asthma, CHD (total), hypertension, mortality (all-cause, DVD), stroke (total), T2DM<br><br>Vegetables<br>Probable: cataract, CVD<br>Possible: hip fracture, stroke, depression, pancreatic disease<br>Limited: CHD, hypertension, mortality (all-cause, CVD)<br>No evidence: T2D	Investigates fruit and vegetables separately only, not combined.<br>Some weaknesses such as protocol missing, not clarified who did what.<br>Not a very recent umbrella review.<br>The included studies are checked, and one study is added below on age-related cataract that was not included in my search. Otherwise, studies included in this review are covered below.
Micha et al 2017 (7)	1 May 2015<br>Meta-analysis of RCTs or prospective cohort studies and two new meta-analyses.<br>n=23<br><br>Fruit and vegetables are based on three meta-analyses: Gan 2015	Dietary factors (foods and nutrients)	CVD<br>CHD<br>Stroke<br>Diabetes	Low intake of fruit and vegetables (and also other foods) were identified as having an etiological protective effect on coronary heart disease and stroke, with probable or convincing evidence using Bradford-Hill criteria.	Assessment of etiologic effects of foods and nutrients and optimal intakes. Some important and more comprehensive meta-analyses on CVD and mortality have been published since May 2015 though.

	below regarding, plus de novo meta-analysis				
Deng et al 2016 (8)	September 2015<br>n=3 for fruits and vegetables	Food groups	Stroke risk		This article is covered by Aune

CVD

**Author** year	**Scope of the SR/MA (search date and number of studies included)**	**Exposures**	**Outcomes**	**Results (SR/MA)**	**Quality/comments***
**Sun et al (2022) (9)**	10 Feb 2022<br>Prospective studies<br>n=28 for fruit<br>n=6 for 100 % fruit juice	Fructose containing food sources, including fruits and fruit juice	CVD incidence and mortality	MA: Reduced risk reductions seen for fruit intake and CVD, CHD and stroke. Dose-dependent association with a threshold of 400 g/d.	High quality. Is now included in the text, but only include fruit and not focused on fruit and vegetables, so not included in table 1.
Bhandari et al 2023 (10)	Jan 2022<br>Prospective studies with repeated measurements of diet (hence the low number of studies)<br>n=3 for fruits and vegetables	Ten food groups, including fruit and vegetables	CVD mortality	MA: Reduced risk reduction (0.7). However, only three studies of which one on apples only, one on vegetables and one on fruits.	Few studies.
**Zurbau et al 2020 (11)**	June 2019<br>Prospective studies<br>n=81 cohorts<br>(117 studies)	Fruits and vegetables separately and combined.<br>Different types of fruits and vegetables, including berries and fruit juice and dried fruits (potatoes not analysed).<br>Highest vs lowest (not dose-response)	CVD, CHD and stroke incidence and mortality separately	MA: Risk reductions shown for both fruits and vegetables and fruits and vegetables combined for all outcomes.<br>Beneficial effects from some fruits and vegetables.	A2: High (11)
**Bechthold et al 2017 (12)**	March 2017<br>Prospective studies (cohort, case-control, follow-up of RCTs)<br>n=19 for vegetables (cohorts)<br>n=17 for fruits (cohorts)	Twelve food groups, including fruits and vegetables dose-response	CVD total and CHD, stroke, heart failure (incidence or mortality, but not clearly stated)	MA: Risk reductions for both fruit and vegetables (not studied combined). Moderate or low quality of evidence.	A2: High (12; and >13 according to Yip, but Aune was selected by Yip instead), a comment regarding change of protocol. Not primarily focused on fruit and vegetables.

**Aune et al 2017 (13)**	29 September 2016<br>Prospective studies<br>n=95 cohort studies<br>(142 publications)	Fruits and vegetables separately and combined.<br>Dose-response.<br>Different types of fruits and vegetables including berries, fruit juice, dried fruits and potatoes.	Incidence or mortality from:<br>CHD<br>Stroke<br>Total CVD<br>Total cancer<br>All-cause mortality	MA: Risk reductions shown for both fruits and vegetables and fruits and vegetables combined for all outcomes.<br>Beneficial effects from some fruits and vegetables.	A2: High (11.5 and 12.5 according to Yip)
Zhan et al 2017 (14)	June 2014<br>Prospective cohort studies<br>n=38 studies<br>(47 cohorts)	Fruits and vegetables separately and combined.	CVD mortality and incidence	MA: Risk reductions shown for both fruits and vegetables and fruits and vegetables combined for all outcomes.	Should be covered by Zurbau. Not fully evaluated, but no published protocol, no clear search strategy.
Lippi et al 2016 (15)	Date not stated.<br>Prospective and case-control<br>n=4 prospective<br>n=1 case-control	Vegetable intake	Venous thromboemolism.	SR: No clear support of a protective effect.	Less relevant outcome. Very short method section, no exclusion/inclusion criteria e.g., or method for quality evaluation.
Gan et al 2015 (16)	July 2014<br>Prospective cohort studies<br>n=23	Fruits and vegetables separately and combined<br>Dose-response	CHD risk	MA: Risk reductions shown for both fruits and vegetables and fruits and vegetables combined.	Should be covered by Aune.
Hu et al 2014 (17)	January 2014<br>Prospective cohort studies<br>n=24 studies<br>(20 cohorts)	Fruits and vegetables separately and combined.<br>Dose-response	Stroke	MA: Reduced risks.	Should be covered by Aune and Zurbau.
Wang et al 2014 (18)	30 Aug 2013<br>Prospective cohort studies<br>n=16	Fruits and vegetables separately and combined.<br>Dose-response	Risk of all-cause, cancer and CVD mortality (not incidence)	MA: Risk reductions shown for both fruits and vegetables and fruits and vegetables combined for all outcomes (approx. 5%)	Should be covered by Zurbau. Is also commented upon in Aunes introduction. A2: 13 according to Yip.
Sherzai et al 2012 (19)	Not stated<br>Epidemiologic studies<br>n=34	Dietary patterns as well as food groups, incl. fruit and vegetables	Risk of stroke	MA: Protective effect from fruit and vegetables	Should be covered by Aune and Zurbau.

Total mortality

**Wang et al 2021 (20)**	Sept 2018<br>Prospective studies	Fruit and vegetables	All-cause mortality	Lowest risk reduction at around	A2: High (12)

	n=24 cohorts<br><br>+ Analysis of NHS and HPFS regarding cause-specific mortality and subgroups of fruits and vegetables	(subtypes only for the 2 US cohorts)		5 servings of fruit and vegetables (23 % risk reduction)<br>2 servings of fruit<br>3 servings of vegetables<br>Minimal risk reduction above this intake.	Comments on Aune, not Zurbau regarding the 2 cohorts and analysis of subgroups of fruit and vegetables.
**Schwingshackl et al (21) 2017**	31 Dec 2016<br>Prospective studies<br>n=37 for vegetables<br>n=34 for fruit	Several food groups, including fruits and vegetables.<br>Dose-response	All-cause mortality	MA: Risk reductions shown for both fruits and vegetables.	A2: Same protocol and methods as Bechthold above.
**Aune et al 2017 (13)**	29 Sep 2016<br>Prospective studies<br>n=95 studies<br>(142 publications)	Fruit and vegetable consumption.<br>Dose-response<br>Different types of fruit and vegetables, including potatoes	Incidence or mortality from:<br>CHD<br>Stroke<br>Total CVD<br>Total cancer<br>All-cause mortality	MA: Risk reductions shown for both fruits and vegetables and fruits and vegetables combined for all outcomes.<br>Beneficial effects from some fruits and vegetables (including potatoes)	A2: High, same comments as above.
Aasheim et al (22)<br>*Found in Yip*	Three cohorts in UK (from EPIC)<br>Adults	Tinned fruit	Mortality	Increased risk (approx. 10 %) for all-cause mortality.	Not a systematic review but supports the conclusion in Aune.
Wang et al 2014 (18)	30 Aug 2013<br>Prospective cohort studies<br>n=16	Fruits and vegetables separately and combined.<br>Dose-response	Risk of all-cause, cancer and CVD mortality (not incidence)	MA: Risk reductions shown for both fruits and vegetables and fruits and vegetables combined for all outcomes (approx. 5%)	Should be covered by Zurbau. Is also commented upon in Aunes introduction. A2: 13 according to Yip.

T2D

**Halvorsen et al 2021 (23)**	20 October 2020<br>Prospective cohort studies, case-control, nested case-control<br>Mostly adults, some studies include younger people<br>n=23	Fruits and vegetables separately and combined.<br>Also types of fruits and vegetables, including berries, potatoes and fruit juice.	T2D risk	MA: Weak inverse association between fruit and vegetables intake and risk of T2D. Unclear results for individual foods.	A2: High (12)<br>This seems to be the best and most comprehensive and updated review regarding T2D, investigating both fruits and vegetables and their subgroups.
Chen et al 2018 (24)	Singapore Chinese health study<br>Including a meta-analysis of prospective studies on green leafy and cruciferous vegetables<br>n=11	Green leafy and cruciferous vegetables	T2D risk	MA: Protective effect of green leafy vegetables or cruciferous vegetables, but borderline significance.	Focused only on some specific fruit and vegetables.<br><br>(Both a study on Asian population and a meta-analysis)<br><br>Should be covered by Halvorsen.
Schwingshackl et al 2017 (25)	February 2017<br>(cohort, case-control, follow-up of RCTs)	Twelve food groups, including fruits and vegetables.	T2D risk	MA: Reduced risk for fruits and vegetables separately, approx. 10 %	A2: High (12; and >13 according to Yip, but Wu selected instead)<br>Should be covered by Halvorsen.

	n=13 for vegetables<br>n=15 for fruit	Dose-response		at 200-300 gram each, no risk reduction after that.	
Jia et al<br>2016 (26)	December 2014<br>Prospective cohort studies<br>n=5 articles<br>(7 cohort studies)	Citrus and cruciferous vegetables	T2D risk	MA: Reduced risk for cruciferous vegetables not citrus fruit.	Focused only on some specific fruit and vegetables. Should be covered by Halvorsen.
Wang P-Y et al<br>2016 (27)	21 July 2014<br>Prospective cohort studies<br>n=15 for fruit and vegetables	Fruits and vegetables separately and combined, and their fibre. Also types of fruits and vegetables.	T2D risk	MA: Reduced risk for fruit. Non-significant reduced risk for vegetables and F&V combined. Also reduced risks for blueberries, green leafy vegetables, cruciferous vegetables and yellow vegetables.	Should be covered by Halvorsen.
Wu et al<br>2015 (28)	June 2014<br>Prospective cohort studies<br>n=7 for fruits and vegetables<br>n=7 for vegetables<br>n=9 for fruit (7 articles)	Fruits and vegetables separately and combined<br>Dose-response	T2D risk	MA: Reduced risk for both fruits (2 servings/day) and vegetables (2-3 servings/day), no risk reduction at higher levels.	A2: 11 according to Yip and selected by Yip instead of Schwingshackl.<br>Should be covered by Halvorsen.
Li et al<br>2014 (29)	February 2014<br>Prospective cohort studies<br>n=10 articles<br>(13 comparisons)	Fruits and vegetables separately and combined<br>Green leafy vegetables (not clearly stated in the aim)<br>Dose-response	T2D risk	MA: Reduced risk for fruit intake and vegetables intakes (10%, high vs low for both, not significant for vegetables). Trend for fruits and vegetables combined. No reduction at higher risks. Reduced risk for green leafy vegetables.	Should be covered by Halvorsen.
Li et al<br>2015 (30)	4 November 2013<br>Prospective cohort studies<br>n=7 studies<br>(9 cohort studies)	Fruit intake only	T2D risk	MA: Reduced risk. No reduced risk above 200 g/d.	Should be covered by Halvorsen.
**Other**					
Muraki et al<br>2013 (31)	Health professional studies (3 cohorts)	Individual fruits, including fruit juice	T2D risk	Greater consumption of e.g. blueberries, grapes and apples reduced the risk.<br>Fruit juice was associated with higher risk.	Not a systematic review
Cooper et al<br>2012 (32)	The EPIC study	Fruits and vegetables separately and combined.<br>Also types of fruits and vegetables	T2D risk	Weak protective effect from fruit and vegetable intake in general. Root vegetables and green leafy vegetables may be protective.	Based on EPIC-cohorts, not completely relevant.

Gestational diabetes

Machairiotis et al 2021 (33)	May 2020, but only from 2019, as an update?<br>Cohort studies<br>n=28	Nutrients and dietary patterns, including foods	Gestational diabetes risk	SR: Mostly dietary patterns. One study on fruits and vegetables shows protective assocations.	Too few studies on fruits and vegetables. Not primarily focused on fruits and vegetables.
Monammadi et al 2020 (34)	Jan 2018<br>Cohort studies<br>n=5	Fruit intake	Gestational diabetes risk	MA: High fruit intake associated with 5% lower risk	Only fruit is investigated. Maybe enough to report about T2D.
Mijatovic-Vukas et al<br>2018 (35)	2 February 2017<br>Longitudinal studies<br>n=40	Diet and physical activity, including carbohydrates (fruit, fibre, potato)	Gestational diabetes risk	SR: High fruit intake not a risk (based on one study only). Fruit fibre protective (based on one study only)<br>MA only made for physical activity.	Too few studies on fruits and vegetables. Not primarily focused on fruits and vegetables.
Schoenaker et al 2016 (36)	January 2015<br>Observational studies<br>n=10 cohorts<br>n=6 cross-sectional<br>n=5 case-control	Dietary intake, including energy, nutrients foods and dietary patterns	Gestational diabetes risk	SR: No significant results for most foods or food groups, but not primarily looked for.	No significant result, but fruits and vegetables not primarily looked for.

Hypertension

**Schwingshackl et al<br>2017 (37)**<br>ERRATUM 2018	June 2017<br>Cohort, case-control, follow-ups of RCTs<br>Adults<br>n=8 for vegetables<br>n=7 for fruits	Twelve food groups, including fruits and vegetables<br>Dose-response	Risk of hypertension	MA: Inverse associations for fruits (small non-significant trend for vegetables). Low quality of evidence.	A2: Same protocol and methods as the other by the same author.<br>14 according to Yip
**Wu et al<br>2016 (38)**	4 November 2015<br>Prospective cohort studies<br>Adults<br>n=7 studies (9 cohorts)	Fruits and vegetables separately and combined.<br>Dose-response	Risk of hypertension	MA: Inverse association between intake of fruit and/or vegetables comparing high and low. Dose-response was found for fruit and vegetables combined.	A2: High (13)<br><br>Partly covered by Schwingshackl, but also include fruits and vegetables combined.
Li B<br>2016 (39)	April 2015<br>Cohort, case-control, cross-sectional<br>n=23 articles (25 studies)<br>n=3 cohorts<br>n=2 case-control<br>n=20 cross-sectional	Fruits and vegetables separately and combined.	Hypertension	MA: Inverse associations for fruit and fruits and vegetables combined (not for vegetables)	Mainly based on cross-sectional studies.
Kalyoncu et al 2014 (40)	25 June 2013<br>Observational and interventional studies in youth (10-24 years)<br>n=9	Nutrition factors	Prevention of hypertension	SR: Results indicate that fruits and vegetables have preventive effects later in life (among other types of foods), but this is based only on one study from 2010 (the other studied other nutritional aspects or	Results regarding fruit and vegetables (not primarily focused on) are based on only one study from 2010.

				combination of other health promoting activities).	

CVD risk factors and systemic inflammation

**Observational**					
Collese et al 2017 (41)	December 2015<br>Cross-sectional studies and cohort studies<br>Adolescents<br>n=11 cross-sectional<br>n=1 cohort	Fruits and vegetables separately and combined.<br>Including fruit juice	CVD risk factors	SR: Inconsistent results, insufficient studies to conclude.	Almost only cross-sectional studies.
**Mixed**					
Adegbola et al (2022) (42)	31 Dec 2019<br>RCTs<br>Cohorts,<br>Case-control<br>Cross-sectional<br>Patients at risk of PAD and patients with PAD	Nutrition, including fruit and vegetables	Peripheral artery disease, primary and secondary prevention	SR: Primary prevention: n=3 cohort studies, 1 showed an association, 2 showed no association	Few studies, not a main outcome, therefore not included.
**Hosseini et al 2018 (43)**	March 2018<br>Cohort, case-control, RCTs<br>Adults, adolescents and children<br>n=83 in total<br>n=71 RCTs<br>n=10 cross-sectional<br>n=2 cohort	Fruits and vegetables separately and combined, including juices and extracts	Inflammatory biomarkers<br>Immune cell populations	MA: Analysis based on RCTs:<br>Inverse associations between fruit and vegetables and inflammation.<br>Less clear results for immune response.<br>Interventions mostly based on specific juices or extracts of fruit and vegetables.<br><br>Only two studies on children and adolescents.	A2: Medium (10.5)<br>Causes of heterogeneity is not analysed or discussed.<br><br>Interventions mostly based on specific juices or extracts of fruit and vegetables.
Kodama et al 2018 (44)	February 2017<br>Cross-sectional or longitudinal design (intervention studies)<br>Adults<br>n=5 cross-sectional, basis for meta-analysis<br>n=2 interventions	Fruit separately from vegetables	Triglycerides	MA: Fruit intake inversely related to hypertriglyceridemia. No association for vegetables.	A2: Not fully evaluated. Results mostly based on cross-sectional studies.
**RCTs**					
Lee et al 2020 (45)	September 2018<br>RCTs<br>Adults<br>n=1 for vegetables (spinach)	Foods and food ingredients, incl. fruit and vegetables	Acute postprandial triglyceride response	MA: No significant changes in these two specific studies.	Too few studies regarding fruit (fruit juice) and vegetables (spinach).

	n=1 for fruit (orange and orange juice)				
**Hartley et al 2013 (46)** (	Sep-Oct 2012<br>Adults, healthy or at high risk of CVD<br>RCTs<br>n=10<br>n=6 provision of fruits and vegetables,<br>n=4 advice on fruits and vegetables	Advice to increase or provision of fruit and vegetables.	Primary prevention of CVD, events and mortality, or risk factors of CVD (blood pressure, blood lipids, T2D)	MA: No clinical events due to short-term follow up. Short term and heterogenous. Five of the six studies that provided fruit and vegetables only provided one fruit or vegetable. No strong evidence from these studies. Some evidence that advice is beneficial, but this is based only on two trials.	A2: High.<br>A Cochrane study.
**Other**					
Schwingshackl et al<br>2018 (47)	January 2018<br>RCTs<br>n=66 (86 reports)	11 food groups including fruit and vegetables	Cardiometabolic risk factors	MA: Nuts, legumes and whole grains more effective than other food groups. Fruit and vegetables were ranked best for SBP reduction.	A network meta-analysis and not specifically a summary of RCTs regarding fruit and vegetables.

Metabolic syndrome

**Observational**					
**Lee et al 2019 (48)**	October 2018<br>Observational studies (cross-sectional, case-control, cohort)<br>Adults (but not specified)<br>n=7 cross-sectional<br>n=2 cohort	Fruits and vegetables separately.<br>Dose-response.	Metabolic syndrome	MA: Inverse associations for fruit but not for vegetables. Highest vs lowest as well as dose-response.	A2: Not fully evaluated, seems ok.<br>Mostly cross-sectional (8 of 10)
**Zhang and Zhang 2018 (49)**	September 2017<br>Observational studies<br>Mixed ages<br>n=20 cross-sectional<br>n=1 case-control<br>n=5 cohorts	Fruits and vegetables separately and combined.	Metabolic syndrome	MA: Inverse associations for fruits and vegetables separately and combined. Highest vs lowest.<br><br>Only significant for vegetables when looking at cohorts only (n=3 for vegetables, n=2 for fruit, n=2 for fruit and vegetables)<br><br>Inverse associations for both fruits and vegetables seen also in adolescents separately.	A2: Not fully evaluated, seems ok.<br>13 according to Yip.<br>Mostly cross-sectional studies:<br>13 of 16 for vegetables<br>14 of 16 for fruit<br>7 of 9 for F&V
Tian et al<br>2017 (50)	July 2017<br>Observational studies adolescents and adults<br>n=13 cross-sectional<br>n=3 cohort	Fruits and vegetables separately and combined.	Metabolic syndrome	MA: Inverse associations for fruits and vegetables separately and combined	Mostly cross-sectional studies. Should be covered by the two later studies.

**RCTs**					
Shin et al<br>2015 (51)	10 December 2013<br>RCTs only<br>Adult metabolic syndrome patients<br>n=9	Fruits and vegetables separately and combined.	Metabolic syndrome components	MA: Inverse associations for diastolic BP.	RCTs on patients.<br><br>Partly covered by Schwingschackl regarding CVD-risk factors, or similar conclusion.<br><br>Not new.

Body weight

**Observational**					
Poorolajal et al<br>2020 (52)	November 2018<br>Observational studies<br>Children and adolescents<br>n=14 studies on F&V, 13 cross-sectional studies and one case-control.	Behavioural factors, including fruits and vegetables consumption (insufficient/sufficient intake)	Risk of childhood obesity	MA: Non-significant decreased risk (but borderline).	A2: Medium-Low (8.5).<br>Methodology seems ok, but short description. Is not primarily focused on fruit and vegetables, but all types of behavioural factors. A detailed description of the studies and how e.g. different amounts of fruit and vegetables in the studies were handled in the meta-analysis seems lacking. No detailed information about confounding factors (only yes/no, three of them were unadjusted) and no discussion regarding the results on fruit and vegetables. Therefore, this study is not included in the NNR summary.
**Schlesinger et al<br>2019 (53)**	August 2018<br>Prospective observational studies (cohort, case-control, follow ups of RCT)<br>Adults ≥ 18 yrs.<br>n= 3 for vegetables and overweight/obesity<br>n=5 for vegetables and weight gain<br>n=4 for fruit and overweight/obesity<br>n=3 for fruit and weight gain	Food groups, incl. F&V separately	Risk of overweight, obesity and weight gain	MA: Intake of vegetables and fruit associated with reduced risk of adiposity (overweight/obesity, abdominal obesity or weight gain)<br>Low or very low quality of evidence.	A2: High (11).<br>This seems to be the most comprehensive review that also included a meta-analysis.

**Nour et al 2018 (54)**	8 October 2018<br>Cohort studies<br>Adults<br>n=10	Vegetable intake only, Studies on potato included in the discussion.	Body weight<br>Weight change<br>Overweight<br>Obesity	SR: Increased intake of vegetables inversely associated with weight-related outcomes in adults.	A2: High (9 of 13). Not mentioned in Schlesinger.<br>Potato is commented upon.<br>Similar conclusions as in Schlesinger 2019, but evidence is considered as moderate instead of low.
Schwingshackl et al 2015 (55)	July 2015<br>Prospective cohort studies<br>n=20 articles (17 studies)<br>14 included in meta-analysis	Fruits and vegetables separately and combined	Change in body weight	MA: Fruit inversely related to weight gain. No changes for F&V combined or for vegetables only. Comparing highest with lowest intakes for all three exposures – lower risk of adiposity.<br>In general, low quality of evidence.	This is commented upon and discussed in Schlesinger, which is more comprehensive.
Ledoux et al 2011 (56)	January 2009<br>Longitudinal or experimental designs child, adolescents or adults<br>n=7 longitudinal studies adults<br>n=4 longitudinal studies children and adolescents	Fruits and vegetables separately and combined	Adiposity	SR: Inverse relationship or mixed results for the longitudinal studies.<br><br>The experimental studies also involved other behavioural changes.	A2: Low (6).<br><br>The studies on adults should be covered by Schlesinger, which is more recent.<br><br>The experimental studies on children also involved other behavioural changes.<br><br>The longitudinal studies on children that showed an inverse association did not control for EE and was of short duration (less than three years). Not new. Therefore, this study is not included in the NNR summary.
**Mixed**					
Guyenet 2019 (57)	Oct 2018<br>RCTs<br>Observational studies<br>n=11 RCTs on body weight<br>n= 5 RCT on energy intake<br>n=25 observational studies	Fresh fruit	Body weight/adiposity<br>Energy intake	SR: No association to adiposity, possible protective.	RCTs: few studies, and not so relevant types of exposures.<br><br>Cohorts: Partly covered by Schlesinger, with focus on weight and weight change. Guyenet includes WC and/or different outcomes of adiposity.
Hebden et al 2017, will be used in fruit juice (58)	15 August 2014<br>Cohort studies or RCTs<br>Healthy adults<br>n=11 RCTs<br>n=6 cohort studies	Fruit only, including fruit juice	Adiposity	SR: Prospective studies:<br>Regarding fruits (n= 5): Whole fruit was associated with reduced risk for long-term weight gain (similar conclusions as Schlesinger above).	A2: Medium (8 of 13)<br>Conclusions from prospective studies regarding fruit should be covered by Schlesinger (similar conclusions).

				Regarding fruit juice (n=1): Fruit juice associated with increased body weight, based on one study (three cohorts from US), more pronounced in people with overweight and obesity.<br><br>RCTs:<br>Regarding fruits (n=7): One study on high fruit diet. The other studied specific fruits (e.g. dried plum, apples, grapefruit, raisins, grapefruit capsule).<br><br>Regarding fruit juice (n=4): Specific juices (grapefruit, xango juice, pomegranate) in overweight people or orange juice in training subjects.	Conclusions from prospective studies regarding fruit juice are based on one study (three cohorts) from US (Pan et al 2013). Is included regarding fruit juice.<br><br>The RCTs seem not so relevant.
**RCTs**					
**Mytton et al 2014<br>Erratum 2017 (59)**	3 September 2013<br>Adults or children, but only adults were found<br>RCTs of increased F&V<br>n=8	Promotion of F&V without specifying other changes in the diet	Change in body weight.<br>Change in energy intake	MA: No support of an effect of F&V on weight gain, possibly a slight positive effect on weight loss.	A2: High.<br>A bit old.
**Kaiser et al 2014 (60)**	1 June 2013<br>RCTs<br>n=2 (fulfilled all criteria)<br>n=5 (fulfilled almost all criteria)	Increased F&V, provided or prescribed	Change in body weight	MA: No support of an effect of F&V for weight loss.	A2: Not fully evaluated.<br>A bit old.
Tapsell et al 2014 (61)	2011 (not specified)<br>Overweight adults<br>RCTs of increased F&V<br>n=16	Vegetable intake only	Change in body weight .	MA: Inconclusive.	A bit old. Should be covered by Mytton and Kaiser.

Bone health

**Brondani et al 2019 (62)**	14 October 2018<br>RCTs and cohort studies<br>Men and women aged >50 years<br>n=13 systematic review<br>n=10 meta-analysis:<br>n=6 cohort studies<br>n=4 RCTs	Fruits and vegetables combined, but also in the form of dietary patterns rich in fruit and vegetables!	Fractures<br>Change in bone markers as secondary outcome	MA of cohorts (n=5): Protective effect on hip-fracture of fruit and vegetables. Level of evidence evaluated as moderate.<br>GRADE: Moderate quality of evidence<br><br>MA of RCTs (n=4): No significant effect on C-terminal telopeptid.<br>GRADE: Low quality of evidence	A2: High (12).<br>However, 3 of the 5 cohorts studied dietary patterns rich in fruit and vegetables, not FV per se. Adjustments are made though, but still unclear if that fully compensate.<br><br>Benetou is included among the studies.

**Hu et al 2018 (63)**	March 2018<br>Postmenopausal women<br>Observational studies in English or Chinese<br>n=11 articles<br>18 studies:<br>n=13 cross-sectional<br>n=5 case-control	Fruits and vegetables separately and combined	Postmenopausal osteoporosis (PMOP) (low BMD measured by DEXA)	MA: Fruit might be beneficial.<br>No significant effect of vegetables.<br><br>For the intake of vegetables and the risk of PMOP, subgroup analysis showed a significant association in case-control studies (OR, 0.62; 95% CI, 0.42–0.90; I2 = 0.0%; FEM), but not in cross-sectional studies (OR, 0.95; 95% CI, 0.69–1.29.	A2: High (13), got the supplement after e-mailing the author (not available otherwise).<br><br>Cross-sectional (mostly) and case-control studies<br><br>The study does not mention Luo et al.
Benetou et al 2016 (64)	Five cohorts in Europe and US<br>Adults aged ≥60 years	Fruit and vegetables intake	Hip fractures	Low intake of fruit and vegetables associated with increased risk compared with moderate intake. More evident among women. High intake not associated with lower risk.	High quality according to Yip. Not a systematic review, but based on a large number of cohorts. Brondani is newer and included Benetous analyses.
Luo et al 2016 (65)	Maj 2015<br>Cohort, case-control, cross-sectional<br>n=5 in total<br>n=1 case-control<br>n=4 cohort studies	F&V separately and combined, and also brassicacae, cruciferae and citrus fruit were specifically searched for.	Hip fractures	MA: Vegetables, and not statistically significant (but a trend) for fruit, reduced the risk of hip fractures.	A2: Medium (10.5)
Hamidi et al 2011 (66)	31 July 2010<br>Women aged >45 yrs<br>Cohort studies and RCTs<br>n=8 total:<br>n=2 RCT<br>n=3 cross-sectional<br>n=1 cohort<br>n=3 case-control<br>n=1 cohort + cross-sectional	F&V separately and combined	Fractures<br>Bone mineral density<br>Bone turnover markers	MA: Little evidence of a protective effect based on studies with low or moderate risk of bias.	Different study designs.<br>A bit old. Should be covered by more recent meta-analyses.

Cognitive disorders/functioning

Nowson et al 2018 (67)	January 2017<br>Cross-sectional, prospective cohort, interventions<br>Elderly >=65 yrs<br>n=2 prospective studies (from 2006 and 2012) and n=5 cross-sectional	Dietary intake, food patterns, including vegetables (not fruit)	Six indices of chronic disease:<br>CVD events (non-fatal)<br>Cognition<br>Mental health<br>Falls and fractures<br>Physical health<br>Frailty	SR: Vegetables associated with reduced risk of cognitive decline, but poor quality and therefore weak evidence. Grade D according to GRADE.<br>No association for non-fatal CVD events or mental health.	A2: Low.

	for vegetables and cognition only				
Mottaghi et al 2018 (68)	30 October 2016<br>Cohort and cross-sectional<br>Older adults (but not specified in inclusion criteria)<br>n=6 studies, 10 effect sizes<br>n=3 cross-sectional<br>n=3 cohort<br>n=1 nested case-control	Fruits and vegetables separately and combined	Cognitive impairment	MA: Protective effect of fruit and vegetables.<br>Only in China and not in Western countries. The results were not affected by study design.	A2: Moderate (6.5)<br><br>Almost same date for the search as Wu, and that article seems better.
**Wu et al 2016 (69)**	13 June 2016<br>Prospective cohort studies<br>Adults (but not specified in inclusion criteria)<br>n=6 cohort studies, 9 comparatives	Fruits and vegetables separately and combined	Cognitive disorders: Alzheimer, dementia<br>Cognitive impairment	MA: Protective effect of fruit and vegetables, 25 % reduction. However, heterogeneity due to ethnic differences and possible publication bias.	A2: High (11.5), but lower according to Yip? Mottaghi was selected by Yip instead of Wu. Comparisons are made with Cao and Loef in the discussion. An extended work of Cao. Lamport is also commented upon. Seems better than Mottaghi.
Cao et al 2015 (70)	1 September 2014<br>Cohort studies<br>Caucasian subjects<br>Adults<br>n=43 in total<br>n=2 for vegetables and fruits	Dietary patterns or food consumption, including food groups.	Dementia (all cause or Alzheimer)<br>Mild cognitive impairment	MA: Tendency towards protective effect on dementia from fruit and vegetables, very wide CI (based on two studies).	Only two studies for fruit and vegetables.
Lamport et al 2014 (71)<br>*Found in Wu et al*	January 2013<br>Observation and intervention studies<br>Adults<br>All but one study sampled older populations (>45 yrs)<br>n=7 longitudinal studies on cognitive function<br>n=8 cross-sectional studies<br>n=6 acute intervention studies on fruit juice	Fruit, vegetables and 100% fruit juice	Cognitive performance (including Alzheimer or dementia)	SR: “Statistically significant benefits of fruit, vegetable, or juice consumption for cognitive performance were observed in 80% (20/25) of the included studies. The limited data from acute interventions indicate that<br>consumption of fruit juices can have immediate benefits for memory function in adults with MCI, although acute benefits have not been observed in healthy adults thus far.”	Does not really follow newer guidelines, but seems to be thoroughly done. No description of extraction of data or quality evaluation of the individual studies, but a long critical evaluation is done.
Loef, Walach 2012 (72)	11 March 2011<br>Cohort studies<br>Adults<br>n=9	F&V separately<br>(and combined?)	Dementia or cognitive decline	SR: Protective effect of vegetables, no evidence for fruit.	Should be covered by Wu.
**Executive functioning**					

Cohen et al 2016 (73)	April 2016<br>All types of studies<br>Children and adolescents (6-18 years)<br>n=21 in total<br>n=4 for fruits and vegetables, all cross-sectional	Healthy diet compared with unhealthy diet, including foods	Executive functioning	SR: Some evidence of a protective effect from fruit and vegetables. Based on 4 cross-sectional studies.	Not specifically focused on fruit and vegetables. Only four cross-sectional studies regarding fruits and vegetables and three of them have not controlled for confounders other than sex, age, ethnicity, socio-economic status and school grades, and none of them have adjusted for other dietary factors, so it may be part of a dietary pattern rather. Not included in the summary.

Mental health

Wang et al 2022 (74)	May 2021<br>Observational studies<br>Children 3-18 years<br>n=22 cross-sectional<br>n=3 case-control<br>n=2 cohort studies	Diet, including food groups	Depressive symptoms	SR: No associations in the cohort studies	Few cohort studies, not specifically investigating fruit and vegetables.
**Matison et al 2021 (75)**	December 2020<br>Cohort studies<br>Middle-aged and older adults<br>n=21 in the meta-analysis<br>n=4 for fruit and vegetables<br>n=1 for citrus fruit and juices	Nutrition: dietary patterns as well as food groups and nutrients	Incidence of depression	MA: Reduced risk of depression for both vegetables and fruit.	A2: High (13.5 or 14)<br><br>Note the restricted age groups, in comparison with Saghafian.
Dabravolskaj et al 2022 (76)	30 October 2020<br>Observational studies<br>Youth 10-18 years<br>n=8 cross-sectional<br>n=1 case-control<br>n=3 cohort	Fruit and vegetables	Common mental disorders (anxiety, depression)	SR: No associations seen in the three cohort studies after adjustments for confounders.	Few cohort studies.
**Dharmayani et al 2021 (77)**	31 August 2020<br>Cohort studies<br>Young people and adults aged 15-45 years<br>n=12	Fruit and vegetables separately and combined	Depressive symptoms	SR: Positive effect of fruit and more inconsistence evidence regarding vegetables, but a possible association is suggested by the authors.	A2: High (10.5 of 13)

Glabska et al 2020 (78)	June 2019<br>Observational studies (cross-sectional, cohort)<br>Adults<br>n=61	Fruit and vegetables separately and combined, including juices	Mental health (mental well-being, quality of life, sleep quality, mood anxiety, depression, etc.)	SR: Positive association in general.	Uncertainty on conflict of interest/funding related aspects. The result section is poorly organized and synthesized. Nevertheless, it is quite extensive and broad.
Glabska et al 2020 (79)<br>(Polish journal)	June 2019<br>Observational studies<br>Adolescents<br>n=17	Fruit and vegetables separately and combined, including juices	Mental health (mental well-being, quality of life, sleep quality, mood anxiety, depression, etc.)	SR: Positive association in general, also for some specific foods.	Mostly cross-sectional.<br>Not clearly stated or discussed.
Guzek et al 2020 (80)<br>(Polish journal)	June 2019<br>Observational studies<br>Children	Fruit and vegetables separately and combined, including juices	Mental health (mental well-being, quality of life, sleep quality, mood anxiety, depression, etc.)	SR: Positive association in general.<br>Exposure mixed with other foods?	Mostly cross-sectional.<br>Not clearly stated or discussed.
Tuck et al 2019 (81)	1 February 2019<br>Prospective studies, RCTs<br>Healthy adults<br>n=10	Vegetables only or fruit and vegetables combined.	Mental health (e.g. depression) or psychological well-being (more than absence of mental health disorders). Risk of suicide was excluded since it is an extreme	SR: Protective effect on psychological well-being. Less clear effect on mental health.<br><br>I think it is difficult to draw the conclusions regarding fruit and vegetables separately. The search strategy was only focused on vegetables, not fruit.	A2: Low (6/13). Conclusions regarding different effects of fruit and vegetables are drawn from three studies, but quality is not considered. Search terms only include vegetables, but they draw conclusions regarding fruit.<br><br>Not mentioned in Matison (but not a MA).
**Saghafian et al 2018 (82)**	October 2017<br>Cohort studies and case-control studies (analysed separately)<br>Adults<br>n=17 in total<br>n=6 cohorts for fruit<br>n=7 cohorts for vegetables	F&V separately and combined.<br>Dose-response	Depression or anxiety	MA: Protective effect of fruit and vegetables separately and combined for depression. Too few studies to investigate anxiety.	A2: Medium (9.5)<br><br>Mentioned in Matison.
Molendijk et al 2018 (83)	6 March 2017<br>Prospective cohort studies<br>n=24<br>n=6 for fruit and 7 for vegetables	Diet quality, including food groups	Depression	MA: Vegetables protective, not fruit, but close to significant.	Should be covered by Saghafian. More focused on diet quality.<br><br>Mentioned in Matison, but only referred to as healthy diets.
Liu et al 2016 (84)	June 2015<br>Cross-sectional and cohort studies<br>Not specified, but includes also younger people<br>n=10 for fruits	Fruit and vegetables separately	Depression	MA: Inverse associations seen, also in the three cohort studies.	A2: Not evaluated but 10 according to Yip. Should be covered by Matison (one study that differs because of restricted age group in Matison).

	n=8 for vegetables<br>n=3 cohort studies<br>n=7 cross-sectional				Mentioned in Matison. Criticized by Saghafian (and others): Not all studies were included, and adjustments were not made.

Wheezing and asthma and allergies

Hosseini et al<br>2017 (85)	June 2016<br>Adults and children<br>n=30 cross sectional<br>n=13 cohort<br>n=8 case-control<br>n=7 experimental	Fruit and vegetables separately and combined	Asthma/wheezing<br>Immune responses	SR:<br>Adults, n=4 cohorts, protective effect on asthma.<br>Children and adolescents, n=9 cohorts, protective effect on asthma is indicated.<br>No clear results regarding pregnant mothers and their child later?<br><br>Experimental studies on children with asthma, little data or no associations.<br><br>MA: Risk of prevalent asthma (also for cohorts). Adults and children are combined in the MA due to too few studies to analyse separately. Most of the studies are cross-sectional.	A2: Medium (9.5) and 9 according to Yip.<br>Most of the studies are cross-sectional.<br><br>Seyedrezazadeh not mentioned, which seems better and therefore included in the summary and not this.
**Seyedrezazadeh et al<br>2015 (86)**	July 2013<br>Prospective, case-control, cross-sectional<br>Adults and children<br>n=12 cohort<br>n=4 case control<br>n=26 cross-sectional	Fruit and vegetables separately and combined	Wheezing and asthma<br>Asthma symptoms	MA: Inverse association with fruit and raw vegetables and wheezing.<br>Modest association for vegetables and asthma.	A2: Medium (8.5)<br>Study quality is not reported or considered. Otherwise, it seems like a better MA than Hosseini, with more sensitivity analysis.
Nurmatov et al<br>2011 (87)	May 2009<br>Cohort, case-control, cross-sectional, interventions<br>Children<br>n=62 in total, for F&V:<br>n=3 cohort<br>n=2 case-control<br>n=17 cross-sectional	Nutrients and foods, including fruit and vegetables	Asthma/wheezing<br>Atopic disorders	MA: Indication that higher consumption during pregnancy and children in early life leads to lower risk of asthma in children. More strongly for fruit than for vegetables.	Mostly cross-sectional<br>A bit old.
**Umbrella review**					
Garcia-Larsen et al<br>2015 (88)	December 2013<br>Systematic reviews<br>n=7	Diet, included food groups	Asthma	Inverse association for fruits.	Too old.

Eye diseases

**Dinu et al 2018 (89)**	January 2018<br>Prospective studies<br>Adults ≥18 yrs.<br>n=26 in total<br>n=4 for vegetables<br>n=3 for fruit	Food groups, included fruit and vegetables	Age-related macular degeneration (occurrence and progression)	MA: Reduced risk for fruit and vegetable, respectively, but of borderline statistical significance.	A2: High (12)<br><br>Only prospective studies (compared with Huang)
Dow et al 2018 (90)	October 2017<br>All types of studies<br>n=27<br>n=13 prospective<br>n=6 RCTs or nested case-controls within RCTs<br>n=10 cross-sectional<br>n =4 case-control	Dietary intake or foods, included fruit and vegetables combined	Diabetic retinopathy	SR: Only two studies on fruit and vegetables: One cohort in Japan found protective effect of high fruit consumption. One cross-sectional study from US found inverse association for high flavonoid fruit and vegetable consumption.	Too few studies.
Huang et al 2015 (91)<br>*Found in Angelinos umbrella review,*	April 2015<br>Prospective, case-control, cross-sectional<br>n=5 cohort<br>n=3 case-control<br>n=1 cross-sectional	Vegetable consumption	Age-related cataract	MA: Inverse association (28% high versus low). Also found in cohort studies only. In America and Europe only.	A2: Medium (8). Risk of bias is not included, but all other steps seem ok and clear description of confounders. Stratified analyses are done for study design.Only vegetables, not fruit.

Periodontal diseases

**O'Conner et al 2020 (92)**	September 2018<br>Older adults ≥60 yrs<br>Cross-sectional, longitudinal<br>n=3 cross-sectional<br>n=6 longitudinal, 2 for fruit and vegetables in Japan	Diet and nutrient intake, including fruit and vegetables and other food groups, also types of fruit and vegetables	Periodontal diseases	SR: Reduced risk of periodontal disease for fruit and vegetables (two studies), especially dark green and yellow vegetables (one of the studies), both from Japan.	A2: High (9 of 13)<br>Few studies. This is commented upon in the summary.<br>Not primarily focused on fruit and vegetables.
Skoczek-Rubinska et al 2018 (93)	15 December 2017<br>Cohort, interventions, RCTs, cross-sectional<br>All ages<br>n=15 in total<br>n=4 interventions<br>n=1 retrospective cohort	F&V separately and combined, including types of fruit and vegetables<br>(fruit juice excluded)	Periodontal diseases	SR: Fruit and vegetables (especially some fruit and vegetables) may prevent the progression of periodontal diseases.<br><br>1 study on children<br>15 studies on adults	A2: Low (6). A discussion or information on confounders is lacking. The two cohort studies are included in O'Conner. The retrospective study only looks at guava fruit intake. The intervention studies look at

	n=2 prospective cohort<br>n=8 cross-sectional			9 studies on elderly (≥65 yrs)	specific exposures, not fruit and vegetables in general. The rest are cross-sectional studies.

Inflammatory bowel disease

**Milajerdi et al 2021 (94)**	January 2019<br>Prospective cohort studies or nested case-control studies<br>Adults<br>n=6 in total<br>n=4 for fruit<br>n=3 for vegetables	Dietary fiber, fruit and vegetable intake	Ulcerative colitis and Crohn’s’ disease)	MA: Protective effect of both fruit and vegetables on ulcerative colitis and Crohn’s’ disease	A2: Medium/low (8)
**Li et al 2015 (95)**	15 September 2014<br>All types of studies<br>All ages<br>n=14 case-control	F&V separately and combined	Ulcerative colitis and Crohn’s’ disease	MA: F&V might be inversely associated with risk of ulcerative colitis and Crohn’s’ disease	A2: Medium (8.5) but 12.5 according to Yip.<br>Only case-control studies.
Hou et al 2011 (96)	August 2010<br>Cohort and case-control<br>n=18 case-control<br>n=1 cohort	Dietary intake, including food groups and nutrients	Ulcerative colitis and Crohn’s’ disease	SR: Fruit lowered the risk of Crohn’s’ disease (n=5)<br>No significant association for ulcerative colitis (n=8), but lower risk	Mostly case-control studies, only one cohort.

Frailty and muscle function

**Ghoreishy et al 2021 (97)**	February 2021<br>Prospective observational or cross-sectional<br>Adults ≥ 50 years old<br>n=14 in total<br>n=10 cohorts<br>n=4 cross-sectional	F&V separately and combined.<br>Dose-response	Frailty	MA: Reduced risk of frailty based on cohort studies. Dose-response association. More clearly for fruit than for vegetables.	A2: High (11)<br><br>(Unclear statement in the intro about a systematic review that is not a systematic review.)<br><br>Granic and Feng not mentioned.
Kojima et al 2022 (98)	January 2021<br>Middle-aged and older people<br>n=5 cohorts in total	Fruit and vegetables separately and combined	Incident frailty	MA:<br>Based on 4 cohorts.<br>Inverse association (high vs low) for fruit and vegetables combined as well as for vegetables alone. No association for fruit (broad CI).	A2: Critically low according to modified Amstar-2
**Granic et al 2020 (99)**	5 March 2020<br>Observational and intervention studies.<br>Adults ≥ 50 years old	Type of whole food, included fruit and vegetables (included juices)	Muscle mass<br>Muscle function<br>Sarcopenia	SR: High intake of fruit and vegetables (separate and together) associated with better muscle strength and function in observational	A2: High (10 of 13)

	For fruit and vegetables:<br>n=3 prospective studies<br>n=2 cross-sectional<br>n=9 intervention studies (check again)			studies (n=3 only) but scarce evidence from intervention studies. Moderate evidence for the role of fruit and vegetables regarding muscle strength and sarcopenia.	Lack info of and a discussion of confounding,but RoB is clearly considered.
Feng et al 2017 (100)	September 2016<br>Longitudinal studies<br>Adults >60 years<br>n=23	Risk factors in general, not specifically diet, lifestyle factors.	Frailty	Inverse association for fruit/vegetables intake – only one study!	Not primarily focused on fruits and vegetables – only one study.

Other outcomes

Huo J et al 2022 (101) *Added in updated search 3*	July 2022<br>RCTs<br>n=11	Fruit	Functional constipation	MA: Fruit intake may have a beneficial effect on stool consistency, stool frequency, gut and microbiota	Only fruit is investigated. Not included since not a major health outcome
Lee et al 2022 (102) *Added in updated search 3*	Jan 2022<br>RCTs<br><br>Fruit<br>n=1 substitution trials (IHCL)<br>n=1 substitution trials (ALT)<br>n=4 addition trials (ALT)<br>n=2 addition trials (AST)<br><br>Fruit juice:<br>n=2 substitution trials (ALT)<br>n=7 addition trials (ALT)<br>n=2 substitution trials (AST)	Sources of fructose, including fruit and fruit juice	Non-alcoholic fatty liver disease, different markers	MA: Some evidence that excess energy from SSB increase liver fat. The role of other sources is uncertain.	Not focused on fruit and fruit juice, but on fructose-containing sugars. No evidence regarding fruit or fruit juice. Not a major health outcome.
Bäcklund R et al 2023 (103)	Nov 2021<br>Observational studies<br>n=2 case-control<br>n=3 cohort	Diet, including fruit and vegetables	Rheumatoid arthritis	SR: Mixed, not conclusive (tendency toward protection?)	Few studies and few prospective studies
**Zhai H et al 2020 (104)**	Not stated<br>Observational studies, all types<br>n=2 case-control<br>n=2 cross-sectional	Fruit and vegetables	Chronic obstructive pulmonary disease	MA:<br>Cohort studies only (dose-response based on 2 cohort and 2 case-control though):<br>Fruit and vegetables:<br>Inverse association (high vs low).<br>No analysis of dose-response.	A2: High (13.5)<br><br>Based on 4 cohorts though.

	n=3 cohort (4, since one included 2 different cohorts)			No heterogeneity<br>Fruit:<br>Inverse association, also significant non-linear dose-response<br>Heterogeneity in relation to region?<br>Vegetables:<br>Inverse association of borderline significance, no significant dose-response.<br>Heterogeneity in relation to region?	
Valera-Gran 2022 (105)	June 2021<br>Children and adolescents 2-18 years<br>n=5 cross-sectional	Foods, nutrients, dietary patterns	Telomere length	SR: Indications of positive association between fruit and vegetables and telomer length	No prospective studies, fruit and vegetables not specifically investigated
He et al 2020 (106)	Not stated<br>Observational studies<br><br>Fruit:<br>n=6 cross-sectional<br>n=2 case-control<br><br>Vegetables:<br>n=5 cross-sectional<br>n=2 case-control	Food groups	Non-alcoholic fatty liver disease	MA: No ass	No prospective studies. Not a major health outcome.
Takagi et al 2020 (107)	July 2019<br>Observational studies<br>n=3 cohort	Fruit and vegetables	Abdominal aortic aneurysm	MA: Protective effect from fruit, not from vegetables	Research letter
**Lin et al 2020 (108)**	May 2019<br>RCTs or observational studies<br><br>Observational studies<br>n=6 (fruit)<br>n=4 (vegetables)<br>8 cohort studies<br>2 case-control	Dietary and lifestyle factors	Nephrolithiasis (kidney stone)	MA: Protective effect	High confidence according to modified AMSTAR2. Not specifically investigating fruit and vegetables.
Galié et al 2020 (109)	Feb 2019<br>Observational studies<br>RCTs<br>For fruit and vegetables:<br>n=4 case-control<br>n=13 cross-sectional<br>n=1 cohort	Nutrition, including food groups	Telomere health	SR: 1 cohort study – no association	Too few prospective studies

Bajalan et al<br>2019 (110)	April 2018<br>Observational studies, all types<br>n=38 in total<br>n= 4 for fruit and vegetables	Nutritional factors	Dysmenorrhea	SR: Two of the three cross-sectional studies showed inverse association between fruit and vegetables intake and menstrual pain. The case-control study showed no association.	No prospective study found for fruit and vegetables, only three cross-sectional and one case-control. Very limited evidence.
**Zhang et al<br>2019 (111)**	March 2018<br>Observational studies<br>Adults (mainly)<br>n=9 cohort<br>n=4 case-control<br>n=1 cross-sectional	Fruit and vegetables separately	Gallstone disease	MA: Reduced risk from fruit and vegetables. However, clear heterogeneity and mix of case-control and cohort studies.	A2: Medium<br>Based on 9 cohort studies and is a study on fruit and vegetables specifically. Therefore, it is included in the summary.
Salas-Huetos et al<br>2017 (112)	November 2016<br>Case-control, cross-sectional, prospective studies (not RCTs)<br>n=35 in total	Dietary patterns, dietary components, and nutrients	Male fertility parameters and fecundability	SR: Fruits and vegetables among the food groups that are positively related to sperm quality.	Seems ok quality. Only cross-sectional studies though, except one. Most of them not focused on foods. One prospective, that showed a positive result from fruit.
Rafie et al<br>2017 (113)	November 2015<br>Observational studies (all types)<br>Mixed age-groups and subjects (included cancer patients)<br>n=15 cross-sectional<br>n=3 case-control	Dietary patterns and food groups	Telomere length (biomarker of aging)	SR: 5 of the 13 studies showed significant results. The remaining 8 studies showed no association.<br>n=8 cross-sectional for fruit and vegetables<br>n=3 case-control (or nested case control)<br>n=1 prospective<br>However, the study groups differ a lot regarding age, obesity, disease etc.	Seems ok quality. Only cross-sectional and case-control studies.<br>Not very recent.
Murphy et al<br>2014 (114)	June 2013<br>Pregnant women<br>n=6 cohort<br>n=3 retrospective cohort<br>n=2 case-control	Fruit and vegetables	Infant birth weight or risk for small gestational age births	SR: Limited and inconclusive evidence suggest a positive association between fruit and vegetables and birth weight (protective effect regarding low birth weight or SGA)	A bit old and not a major health outcome. Therefore not included.
Schoenaker et al<br>2014 (115)	May 2014<br>Reproductive-age women<br>n=23 cohorts<br>n=15 cross-sectional	Dietary factors (nutrients, foods, dietary patterns)	Gestational hypertension and pre-eclampsia	SR: Meta-analysis was not done due to too few studies on fruit and vegetables.<br>n=6 case-control<br>n=4 cohort studies<br>Results are presented in additional files, which are not available, so I cannot see the results. Beneficial effects are suggested, but not statistically significant.	Not very recent.
Alsamarrai et al<br>2014 (116)	31 December 2012<br>Prospective population-based studies<br>n=51 in total	Risk factors in general	Pancreatic disease (PD):<br>Acute pancreatitis (AP)	MA: Reduced risk of PD from fruit and vegetables (included cancer)<br>Reduced risk of AP from vegetables (CP not mentioned).	Few studies on fruit and vegetables and this was not the primary focus. No separate analysis for CP.

	n=32 for meta-analysis,<br>n= 26 for cancer<br>n=8 for acute and chronic pancreatitis<br>n= 3 for vegetables<br>n=3 for fruit		Chronic pancreatitis (CP)<br>Pancreatic cancer (PC)	No sign effect on AP from fruit (CP not mentioned)<br>Based on three studies.	A bit old.
**Other**					
Major et al 2018 (117)	Cross-sectional data based on five cohorts in US	Dietary patterns and foods	Serum urate levels (risk factor for gout and also associated with chronic kidney disease, hypertension, metabolic syndrome)	Non-citrus fruits related to lower serum urate levels	Not a systematic review. Only cross-sectional studies from US

Berries

**Observational**					
Guo et al 2016 (118)	January 2016<br>Prospective cohort studies<br>n=5	Berry fruits<br>Anthocyanins<br>Dose-response	T2D risk	MA: 18% risk reduction from berries (high vs low). However, based on the same articles and cohorts as Halvorsen used, but they did not find a significant association.	Should be covered by Halvorsen et al. Halvorsen did not find a significant association and has used has a better methodology, explained by Dagfinn Aune in an e-mail.
**RCTs**					
De Amicis et al 2022 (119)	March 2022<br>RCTs<br>Healthy subjects (adults)<br>n=12	Berries (in any form)	Cognitive function:<br>Attention and concentration<br>Executive functioning<br>Memory<br>Motor skills and construction<br>Processing speed	SR: Usually only one type of berry in each study. No significant effects seen in the majority of studies. However, mixed results.	Modified AMSTAR2: High confidence.
Sweeney et al 2022 (120)	October 2021<br>RCTs<br>n=3	Berries/polyphenols (foods, extracts, suppl)	Gut microbiota<br>Blood pressure	SR: No effects seen	Few studies.
**Bonyadi et al 2022 (121)**	March 2021<br>Middle-aged and elderly healthy subjects or subjects with mild cognitive impairment (MCI)<br>Intervention studies	Whole berries or berry-based products (juice, extract, capsule)	Cognitive components (global cognitive function, psychomotor function, learning and memory, working memory capacity, executive functions	SR: Some indications of protective effects.	A2: High (9.5 of 13)

	n=11 in total (including 1 pilot study)		and brain perfusion/activity)		
Wang Y et al 2023 (122)	Feb 2021<br><br>n=16 (SR)<br>n=11 (MA)	Berry or cherry or citrus fruit or their juice or freeze-dried or powder	Cognition and mood	MA: Some indications that cherry juice may improve psychomotor speed. However, only 2 studies of which one high risk of bias. No associations were found regarding cognitive domains.	Should be covered by Bonyadi, so not included.
**Xu et al 2021 (123)**	Dec 2020<br>Cohort studies<br>RCTs<br>n=44 RCT<br>n=15 cohort	Anthocyanin<br>Anthocyanin-rich berries	CVD markers<br>CVD events	MA: Anthocyanin-rich berries significantly reduced blood cholesterol and C-reactive protein. No effects regarding BMI, blood pressure or endothelial function. RCTs only (no data from cohorts)	Low quality due to lack of discussion on of including studies with low-medium quality in the analysis. However, subgroup analyses are done by study quality (showing no effects in either high quality or low-medium quality studies), but this not discussed.
**Wang Y et al 2021 (124)**	January 2020<br>RCTs<br>Adults<br><br>n=45 trials, of which 38 included in the meta-analysis	Berry or cherry or citrus fruit or their juice or freeze-dried or powder<br><br>Also subgroups of these.	CVD risk factors including BP, endothelial function, blood lipids, inflammatory markers	MA: Significant effect only seen for blood pressure and also for one inflammatory marker.<br><br>Mostly healthy subjects, but some (n=13) with existing CVD risk and a few (n=3) with diagnosed CVD.<br>18 with metabolic syndrome and a few studies with some other diseases	A2: High (also using the modified AMSTAR2).
**Martini et al 2019 (125)**	October 2018<br>RCTs, both acute and chronic interventions<br>Children, adolescents and adults<br>Healthy individuals or patients with CVD risk factors<br>n=22 in total<br>n =13 short-term studies<br>n=11 medium- and long-term studies	Intervention with berries or berry products (raw, juices, supplements)	Vascular function	SR: Positive effect on vascular function is indicated. Particularly effects on flow-mediated dilation and reactive hyperemia index (markers of vascular reactivity) in short term studies and pulse wave velocity and augmentation index (markers of arterial stiffness) in medium- to long-term studies.<br>Type and dose and types of subjects causes of heterogeneity, e.g. No specific results for children.	A2: Medium (8 of 13).<br>High according to modified AMSTAR2
Heneghan et al 2018 (126)	January 2017<br>RCTs<br>Adults<br>n=23	Intake of berries or berry-based product (e.g smoothie, juice), capsules excluded	Markers of cardiovascular and metabolic health:<br>Blood pressure<br>Endothelial function<br>Arterial stiffness<br><br>Blood lipids and blood glucose were secondary outcomes	SR: 13 of the 17 studies of high quality reported a positive effect on at least one marker of cardiovascular risk.<br><br>n=10 for endothelial function<br>n=17 for lipid profile<br>n=6 for blood glucose and insulin or insulin resistance	A2: Medium (7 of 13)<br><br>.

**Luis et al 2018 (127)**	April-June 2016<br>RCTs<br>Adults<br>n=45	Intake of berries or berry-derived products	CVD risk factors (mainly blood lipids and blood pressure)	MA: Favourable effects are seen for lipid profile (reduced total cholesterol, low-density lipoprotein, triglycerids , increased high density lipoprotein) and blood pressure.	A2: medium.
García-Conesa et al 2018 (128)	March 2016<br>RCTs<br>Adults (young to elderly)<br>n=128	Berries, red grapes/wine as sources of anthocyanins (fresh or extracts)	CVD risk factors	MA: Sub analysis showed that berries reduced blood pressure, total cholesterol and increased flow-mediated dilation and reduced glycated haemoglobin.	Not primarily focused on berries, but on berries, red grapes/wine as a group. Some sub analyses are done though.<br><br>Should be covered by Heneghan and Luis (although Heneghan is a SR, not MA)
Huang et al 2016 (129)	August 2015<br>RCTs<br>Adults<br>n=22	Intake of berries (not stated what forms?)	CVD risk factors	Improved blood lipids, blood pressure, glucose, BMI and some other factors. No effect on some other factors.	Not focus on berries but on anthocyanins (from berries). Heneghan and Martini are newer.

Potatoes

Observational					
CVD, all-cause mortality and cancer					
**Darooghegi Mofrad et al 2021 (130)**	August 2020<br>Prospective cohort studies or case-control	Potatoe intake	Site-specific cancer	MA: High vs low and dose-response analyses.<br><br>No association for total cancer, but a sign non-linear dose-response association.<br><br>An association for colorectal cancer, seen only in studies conducted in Europe and in studies not adjusting for BMI. However, an association seen in studies conducted in Europe both for total cancer and some specific cancers (colorectal and prostate).<br><br>No associations seen for different types of potatoes (fried, boiled/mashed/baked/roasted) when analysed separately.	A2: High (12.5)

				Certainty of evidence was considered low or very low using GRADE.	
**Darooghegi Mofrad et al 2020 (131)**	September 2018<br>Prospective cohort studies<br>Qualitative analysis:<br>n=10 all-cause mortality<br>n= 7 for cancer<br>n=3 for CVD<br><br>Meta-analysis:<br>n=10 all-cause mortality<br>n=7 cancer	Intake of potatoes (white, different cooking forms)	Mortality:<br>All-cause<br>Cancer<br>CVD	MA: High vs low and dose-response analyses.<br><br>All-cause mortality:<br>No associations.<br><br>Cancer mortality:<br>No association, but association seen e.g., in long-term studies<br><br>CVD mortality:<br>No meta-analysis done due to too few studies n=3) association.<br><br>Not enough data to analyse fried potatoes.	A2: High (12.5)
**Schwingshackl et al 2019 (132)**	May 2018<br>Cohort, case-control studies, follow-ups of RCTs<br>n=6 for all-cause mortality<br>n=6 for CHD<br>n=6 for stroke<br>n=2 for heart failure<br>n=8 for CRC<br>n=8 for T2D<br>n=4 for hypertension	Intake of potatoes (all types). Also sweet potatoes seem to be included (commented upon in Mofrad 2021)	All-cause mortality<br>CHD<br>Stroke<br>Heart failure<br>T2D<br>Hypertension<br>Colorectal cancer	MA: High vs low and dose-response analysed.<br><br>All-cause mortality:<br>No association total potatoes, subtypes not analysed.<br><br>CHD and stroke:<br>No association for total potatoes, subtypes analysed in one study and no associations.<br><br>Colorectal cancer:<br>No associations for total potatoes. However, stronger associations in European studies and long-term studies. Subtypes not analysed. Should be covered by Mofrad 2021.<br><br>Quality of evidence was low (moderate for fried potatoes).	A2: High (13.5)<br><br>(But Mofrad has comments regarding inclusion of low quality studies.)
Aune et al 2017 (13)	29 sept 2016<br><br>See above in the CVD and all-cause mortality category	*NS= non-significant, BS = borderline significant*<br>*Risk reductions are based on high vs low intake*<br><br>Potatoes<br>CHD: NS, tendency towards protection (n=5, possible heterogeneity)<br>NS dose-response association (n=6)<br>Total stroke: 6% risk reduction, BS (n=4), NS dose-response association (n=4)<br>Ischemic stroke: NS risk reduction (n=5), NS dose-response association (n=5)<br>Heaemorrhagic stroke: NS risk reduction (n=3), NS dose-response association (n=3)<br>CVD: NS risk reduction (n=3), NS dose-response association (n=4)			A2: High<br><br>Should be covered by Schwingschackl and Mofrad above.

		Cancer: NS risk reduction (n=2), NS dose-response association (n=3)<br>All-cause mortality: 22% risk reduction (n=4), BS dose-response association (n=2)			
**Diabetes**					
**Halvorsen et al 2021 (23)**	20 October 2020<br><br>See above in the T2D category.	Fruit and vegetables and subgroups	T2D	MA:<br>Potatoes (table 3)<br>Boiled potatoes: NS risk reduction (n=2), NS dose-response association (n=2)<br>Total potatoes: NS risk increase (n=8), Significant dose-response association (n=8)	A2: High
Guo et al 2021 (133)	August 2020<br>Cohort<br>Case-control<br>Cross-sectional<br><br>T2D<br>n=8 cohort<br>n=1 case-control<br>n=4 cross-sectional<br><br>GDM<br>n=6 cohorts	Potato intake<br>(studies that did not clearly distinguish potatoes from other tubers were excluded)	T2D<br>Gestational diabetes	MA:<br>Analyses of western and eastern populations were done separately. Results for Western pop are given below.<br>Dose-response analyses included both.<br><br>T2D<br>Total potatoes:<br>19% increased risk, supported by linear dose-response assoc.<br>Baked/mashed/boiled:<br>8% increased risk, borderline significant, supported by linear association of borderline sign.<br>Based on three cohort studies in USA only.<br>French fries/fried potatoes<br>33% increased risk, supported by non-linear association.<br>Based on three cohort studies in USA only.<br><br>Most of the studies adjusted for BMI, PA, diet, smoking…<br><br>GDM<br>Total potatoes:<br>Non-significant association (wide CI, tendency towards increased risk). A significant linear dose-response association though.<br>Baked/mashed/boiled:<br>Too few studies to calculate summary estimates for West/East. A significant	A2: Low (according to AMSTAR-NNR2)

				linear dose-response association though.<br>French fries/fried potatoes<br>No significant association.<br>No significant dose-response association, but tendency towards association (wide CI)<br><br>Stronger association in studies with longer follow-up.	
**Quan et al 2021 (134)**	December 2019<br>Cohort studies<br>n=21	Western dietary patterns<br>Foods, including potato, but not specifically F&V	Gestational diabetes	Non-significant relation. But significant risk seen in high-quality studies.	AMSTAR2-NNR: Moderate<br>Should be covered by Guo, but Guo is of lower quality, so this one is included instead.
Quan et al 2022 (135)	September 2019<br>Prospective cohorts<br>n=7	Processed potatoes	T2D	Results indicate a non-significant association (dose-response), especially for French fry	AMSTAR2-NNR: Moderate<br>Should be covered by Guo and Halvorsen, but is included because of the sub-analysis not done by Halvorsen.
Bidel et al 2018	June 2018<br>Cohort studies or clinical trials<br>n=6 cohorts	Potatoes	T2D	Positive association, dose- response.<br><br>Studies in developed countries.	Should be covered Guo and Halvorsen.
Schwingshackl et al 2019 (132), same as above	May 2018<br>See above	Potatoes	T2D	T2D:<br>19 % increased risk for total potatoes (high vs low), also dose-response.<br><br>Subtypes analysis:<br>French fries: 66 % increased risk<br>Boiled/baked/mashed: 9 % increased risk. Stronger associations in studies in USA and long-term studies.	A2: High
**Other outcomes**					
**Borch et al 2016 (136)**	20 October 2015<br>Prospective observational studies or intervention studies<br>n=5 for overweight and obesity<br>n=7 for T2D<br>n=1 for CVD<br>(no intervention studies were identified)	Intake of potatoes (white or yellow only, different cooking forms)	Overweight and obesity<br>T2D<br>CVD<br>Metabolic factors (no results)	SR:<br>Overweight and obesity:<br>Possible association for French fries, otherwise weak evidence.	A2: High (11 of 13)<br><br>T2D and CVD should be covered by the later reports, while overweight and obesity and metabolic factors are not.
**Schwingshackl et al**	May 2018<br>See above	Potatoes	Hypertension	Hypertension:<br>No association for total potatoes (high vs low), but significant dose-	A2: High

**2019 (132)**, same as above				response association, but only for French fries and not for boiled/baked/mashed.<br>An increase in potato intake (total) by 150g per day was positively associated with risk of hypertension.	
**Mixed**					
Åkesson et al 2013 (137)	qSR<br>Prospective observational studies<br>Intervention studies<br><br>n=2 for potatoes	Food groups, included potatoes, berries	Chronic diseases, only one study for potatoes: type 2 diabetes	SR: No conclusions	qSR

Fruit juice

Umbrella reviews					
Fardet et al 2019 (138)	June 2018<br>A systematic review of meta-analysis based on cohort studies and RCTs<br>Children and adults and elderly<br>n=10 in total<br>n=3 for fresh fruits<br>n=1 for canned fruits<br>n=1 for dried fruits<br>n=7 for fruit juices<br>n=1 for sweetened fruit juices	Fruit: fresh and different types of processed fruit (juice, canned etc)	All-cause mortality<br>Chronic diseases (obesity, T2D, CVD, cancer)<br>Metabolic regulation	The prospective studies are covered by Aune and Zurbau and Halvorsen (included fruit juice and dried fruit in Aune and Zurbau).<br><br>The RCTs included are Liu (2013) below and one additional MA not included in my search (Wang 2014, I do not know why), but now added below.<br><br>Summary:<br>Fresh fruits are protective, also dried fruits (based on what?).<br>Unsweetened fruit juice neutral<br>Canned fruit and sweetened fruit juices have negative effects.	The prospective studies are covered by Aune, Zurbau and Halvorsen.<br><br>The MA of RCTs are Liu and Wang (added) below.<br><br>Pan et al is include as a MA, but is a study based on three cohorts from US. Is included in Hebden above and below.
Auerbach et al 2018 (139)	2 February 2017<br>A systematic review of systematic reviews or meta-analysis<br>For outcomes where no systematic review exist, individual studies were briefly reviewed.<br>Children and adults	100% fruit juice	Chronic health conditions (caries, diabetes, glucose control, dyslipidemia, hypertension, liver disease, CVD, body weight)	Mixed picture. Most studies show no associations. Increased risk of caries in children, small increases in long-term weight gain in young children and adults that are likely not clinically relevant.<br><br>Current evidence suggests that there are substantial lower health risks	Regarding glucose, blood lipids and blood pressure: based on Wang and Liu below.<br><br>The other outcomes are based on:<br>Salas 2015 (below)<br>Ravn-Haren 2013 (only apple juice vs apples)

	n=10			from 100% fruit juice compared with SSB.<br><br>Discussion about body weight.	*Hebden (above and below)<br>*O'Neal and Nicklas 2008 (covered by newer MA below)<br>*Crowe-White 2016 (below)<br>*Auerbach 2017 (below)<br>*Xi 2014 (below)<br>*Imamura (below)<br>*Joshipura 1999 (covered by Aune and Zurbau)<br>*Hung 2004 (covered by Aune and Zurbau and Halvorsen)
**SR and MA**					
**Observational**					
CVD, cancer and total mortality					
**Sun et al (2022) (9)**	10 Feb 2022<br>Prospective studies<br>n=28 for fruit<br>n=6 for 100 % fruit juice	Fructose containing food sources, including fruits and 100 % fruit juice	CVD incidence and mortality	MA: No association comparing high vs low (but tendencies towards protection).	High quality
**Kazemi et al (140) 2021**	November 2020<br>Prospective cohort studies<br>n=2 for fruit juice and all-cause mortality<br>n=2 for fruit juice and CVD mortality	Major food sources of fructose: fruit, SSB, yogurt cereals, sweets and fruit juice	CVD mortality<br>Cancer mortality<br>All-cause mortality	MA:<br>CVD:<br>No association (high vs low)<br>All-cause mortality:<br>No association (high vs low) and no dose-response<br><br>Cancer:<br>Too few studies	A2: Moderate confidence<br><br>This is used in the summary instead of Pan.
**Farvid et al (141) 2021**	November 2020<br>Prospective longitudinal studies<br>n=7 for fruit juice	Fruit and vegetables, including subgroups such fruit juice	Breast cancer	MA:<br>4 % increased risk, high vs low.	A2: Moderate
**Li et al 2021 (142)**	Oct 2020<br>Case-control and cohort studies<br>Adults<br>For fruit juice:<br>n=11 cohorts<br>n=6 case-control	SSB and fruit juice	Cancer risk and mortality<br><br>Studies on cancer were based specific cancers	MA:<br>No association in case-control studies<br>Positive association in cohort studies: 6 % increased risk (high vs low), supported by linear dose-response association.<br>However, publication bias, hence "poor evidence"<br><br>Not enough studies to analyse cancer mortality specifically.	A2: High (11.5)<br><br>The discussion is mostly focused on SSB, not fruit juice
**Pan et al 2021 (143)**	21 Sept 2020<br>Prospective cohort studies<br>Adults	Soft drink<br>100% fruit juice	CVD mortality<br>Cancer mortality<br>All-cause mortality	MA:<br><br>CVD<br>No dose-response association	A2: High (13.5)<br><br>Different results regarding CVD mortality compared with Kazemi,

	For 100% fruit juice:<br>n=3 for all-cause mortality<br>n= for CVD mortality<br>n=0 for cancer mortality			An increased risk high vs low (n=2).<br><br>All-cause mortality:<br>No dose-response association and no association high vs low (n=2).<br><br>No study on cancer mortality.<br><br>Evidence was considered insufficient.	but Kazemi has one more study. Both are included.
**Llaha et al 2021 (144)**	31 June 2020<br>Case-control studies<br>Cohort studies<br>Total no of studies<br>n=27 cohorts<br>n=37 case-control	Sweet beverages, including fruit juice	Cancer risk for different types of cancer:<br>Fruit juice is analysed for breast, colorectal and prostate	MA:<br>No association for breast and colorectal (n=3 and n=2). The latter also included cross-sectional studies.<br><br>A small increased risk (3%) for prostate (n=4), high vs low. Only cohort studies.<br><br>CDN: Not significant however there tended to be positive associations between fruit juices and breast, colorectal, and pancreatic cancers.<br><br>No meta-analysis is done for total cancer. Two cohorts on obesity-related cancer, showing inconclusive results regarding fruit juice.	A2: Rated as low confidence AMSTAR2-NNR.
**Zurbau et al 2020 (11)**	June 2019<br><br>See above in the CVD category.	Fruit juice<br>CVD incidence: NS risk reduction (n=5) - *only linear model? (fig S38)*<br>CVD mortality: NS 19 % risk reduction (n=1) - *only linear model (fig S53)*<br><br>CHD incidence: NS risk reduction (n=4) – *only linear model? (fig S95)*<br>CHD mortality: BS 13 % risk reduction (n=3) – *not U-shaped (fig S109)*<br><br>Stroke incidence: 18 % risk reduction (n=4) - *only linear model? (fig S152), looks U-shaped*<br>Stroke mortality: 33 % risk reduction (n=2) - *only linear model (fig S166)*<br><br>GRADE was very low for all the above estimations, except for stroke mortality where it was grade low.			A2: High
Aune et al 2017 (13)	29 sept 2016<br><br>See above in the CVD and all-cause mortality category.	*NS= non-significant, BS = borderline significant*<br>*Risk reductions are based on high vs low intake.*<br><br>Fruit juice<br>CHD: 21 % risk reduction (n=2), NS dose-response association (n=3)<br>Total stroke: 33 % risk reduction (n=2), Sign dose-response association (n=2)<br>CVD: NS 33 % risk reduction (n=1), NS dose-response association (n=2)<br>Cancer: NS risk reduction (n=1), NS dose-response association (n=2)<br>All-cause mortality: 13 % risk reduction (n=1), sign dose-response assoc (n=2)			A2: High<br><br>Should be covered by later meta-analyses.

		Citrus fruit juice<br>Total stroke: NS 10 % reduction (n=2), NS dose-response association (n=2)<br>Ischaemic stroke: 35 % risk reduction (n=2), Sign dose-response association (n=2)<br>CVD: NS 12 % risk reduction (n=2), NS dose-response association (n=2)			
T2D					
**Halvorsen et al 2021 (23)**	20 October 2020<br><br>See above in the T2D category	100% fruit juice<br>NS 5 % risk reduction, NS dose-response (possibly tendency) (n=4)<br>- *no clear U-shape*<br><br>Fruit juice<br>9 % increased risk, BS dose-response association (n=8)<br>- *clear linear shape*<br><br>Fruit drinks<br>28 % increased risk, sign dose-response association (n=6)<br>- *clear linear shape*			A2: High
Imamura et al 2015 (145)	February 2014<br>Prospective studies<br>n=17 cohorts	Sugar-sweetened beverages and fruit juice	T2D risk	MA: Positive association for fruit juice, but might involve bias.	Should be covered by Halvorsen.
Bo Xi et al 2014 (146)	10 Dec 2013<br>Prospective cohort studies	100% fruit juice<br>Sugar-sweetened fruit juice	T2D risk	MA: No association for 100% fruit juice (most of them).<br>Adverse association for sugar-sweetened beverages.	Should be covered by Halvorsen.
Body weight					
**Frantsve-Hawlay et al 2017 (147)**	29 March 2016<br>Cohort studies or controlled trials<br>Children < 12 yrs<br>n=1 controlled trial<br>n=37 cohorts	Sugar-containing beverages including 100% fruit juice	Excess weight gain	SR: Results indicate a positive association between 100% fruit juice and total adiposity for children <5 years and no association for children <12 years, but results are more mixed.<br>However, total energy intake may play a role and magnitude of effect is small and clinical relevance unclear.	A2: High (9.5 of 13)<br><br>No access to appendices.<br><br>Auerbach, Hebden or Crowe-White not mentioned.
**Auerbach et al 2017 (148)**	31 December 2015<br>Longitudinal studies<br>Children 1-18 yrs<br>Cohort or RCTs<br>n=8 (all cohort studies)	100% fruit juice	Change in BMI	MA: 100% fruit juice associated with small weight gain (z-score) in children aged 1-6 yrs (not clinically significant) and not associated with weight gain in children aged 7-18 yrs.<br>Similar conclusions as Frantsve-Hawlay.	A2: High (11.5)<br><br><br>Discusses Crowe-White in the introduction and highlights several limitations.
**See Hebden 2017 (58) above**	Adults	Fruit juice	Body weight	SR: Positive association, based on one study (pooled results from three cohorts in US, Pan et al 2013), more	A2: Medium (8 of 13)<br><br>The original study by Pan et al on the pooled analysis is also

**Also Pan et al 2013 (149), which is included in Hebden** (and not included in the total number of identified articles)				pronounced in people with overweight and obesity.	included in the summary instead of Hebden, since this was the only study of relevance.
Crowe-White et al<br>2016 (150)	2013<br>Children 1-18 yrs<br>Types of studies.<br>n=22 for body weight (7 cohorts, 13 cross-sectional, 2 other)<br>n=10 for nutrient intake (only cross-sectional)	100% fruit juice	Body weight status<br>Nutrient intake	No association for 100% fruit juice and body weight status after controlling for energy intake.	A2: Not fully evaluated. Medium according to Auerbach. Covered by Auerbach 2017.
Cardio-metabolic risk factors					
**Semnani-Azad et al<br>2020 (151)**	24 March 2020<br>Prospective cohort studies<br>n=13<br><br>n= 3 for mixed fruit juice<br>n = 2 for 100 % fruit juice<br>n= 4 for fruit	Fructose-containing foods, including fruit and 100% fruit juices	Incident metabolic syndrome	Protective association for fruit (all doses) and 100% fruit juice (U-shape, moderate doses).<br><br>Evidence considered as moderate.<br><br>A similar U-shape association was also seen for mixed fruit juice.	A2: High (12)<br>(same protocol as for Liu 2019 and Ayoub-Charette 2019)<br><br>Not covered by D'Elia.<br><br>*Aslo added in the fruit and vegetables chapter.*
**Liu et al<br>2019 (152)**	13 December 2018<br>Prospective cohort studies<br>n=15 cohorts (26 articles)<br><br>n= 13 for fruit<br>n=2 for 100 % fruit juice<br>n=	Fructose-containing foods, including fruit and fruit-based products such as 100% fruit juice	Incident hypertension	MA: Protective association for fruit and 100% fruit juice. Comparing highest vs lowest 5 % risk reduction, NS. Using 2 cohorts for analysing non-linear dose-response: U-shape association, maximum protection between 50-150 ml. RR risk at 100 ml/d was 3 %, significant. Certainty of evidence graded as low for both exposures.<br><br>No sign association for fruit drinks (but tendency towards negative effect).<br><br>Regarding 100% fruit juice, same two studies are also covered by D'Elia. Liu is not mentioned by D'Elia and Liu presents more extensive analysis.	A2: High (12)<br><br>*Added in the fruit and vegetables chapter.*

Other outcomes					
See Lamport above (71)	January 2013<br>Observation and intervention studies<br>Adults<br>All but one study sampled older populations (>45 yrs)<br>n=7 longitudinal studies on cognitive function<br>n=8 cross-sectional studies<br>n=6 acute intervention studies on fruit juice	Fruit, vegetables and 100% fruit juice	Cognitive performance (including Alzheimer or dementia)	SR: Only two prospective studies (no consistent results). The study that showed a protective effect did not adjust for the intake of other dietary factors.<br><br>Acute intervention studies investigated specific juices (3 grapefruit, 1 blueberry, 1 cranberry, 1 apple, but the latter with no control group). Small studies.	Not included because of too limited evidence regarding prospective studies. Limited evidence from intervention studies. Also, not very new.
**Ayoub-Charette et al 2019 (153)**	13 September 2017<br>Prospective cohort studies<br>n=3	SSB<br>Fruit juices<br>Fruits	Incident gout	Fruit juice and SSB increased the risk of gout (highest vs lowest). Also significant non-linear dose-response association. However, based on two studies that did not differentiate between pure fruit juice and fruit drink.<br>No association for fruit. Very low certainty.	A2: High (13.5)<br><br>But large uncertainties.
Salas et al 2015 (154)	May 2014<br>Cohort and cross-sectional<br>Children and adolescents, aged 8-19 years.<br>n=11 cross-sectional<br>n=2 longitudinal<br>n=7 for fruit drinks	Diet, including fruit and vegetables and fruit juice	Tooth erosion	MA: Frequent consumption of natural fruit juice was associated with 20 % increased risk of tooth erosion (based on 2 longitudinal ad 5 cross -sectional studies). However, this was seen only in relation to inappropriate tooth brushing.	A2: High (11)<br><br>Not specifically focused on fruit juice. No detailed description of confounding factors and no info about frequencies of intakes.<br><br>Covered by Liska below.
**Mixed**					
**D'Elia et al 2021 (155)**	August 2019<br>Prospective studies and RCTs<br>Adults (I assume)<br>n=21 prospective studies<br>n=35 RCTs	100% fruit juice	Prospective studies:<br>CVD events<br>T2D<br>Hypertension<br><br>RCTs:<br>Blood pressure<br>Lipid profile<br>Glucose homeostasis.<br>Body weight<br>Vascular function	MA:<br>Prospective studies: Inverse association between low-moderate intake and risk of stroke or total CV events. No association for CHD and diabetes risk. Similar conclusions as Aune, Zurbau and Halvorsen.<br><br>D'Elia presents J-shape associations, which are not clearly presented by Aune, Zurbau and Halvorsen.<br><br>Hypertension – only two studies (Auerbach and Duffey, same as in Liu	A2: High (14)<br><br>Prospective studies on CVD and diabetes should be covered by Aune, Zurbau and Halvorsen Pan only looked at mortality.<br><br>Similar conclusions.<br><br>For hypertension, Liu 2018 seems better (is not commented upon by D'Elia).<br><br>Also added in the fruit and vegetables chapter.

				et al 2018, above which seems better)<br><br>RCTs: Favourable effect on blood pressure, arterial compliance and endothelial function. Neutral effects on body weight, blood lipids and glucose metabolism.	
**Liska et al 2019 (156)**	May 2018<br>Cohort or RCTs<br>Adults and children<br>n=5 cohorts in children or adolescents<br>n=9 RCTs on adults	100% fruit juice	Dental health:<br>caries or tooth erosion	SR: No associations in cohorts (children and adolescents)<br>RCTs suggest that fruit juice could contribute to tooth erosion and caries; however methodological uncertainties.	A2: High (11.5 of 13), but no statements of funding/conflict of interest.<br><br>Discusses Salas.
Blanch et al 2015 (157)	1 June 2014<br>n=4 cross-sectional or cohort studies<br>n=42 intervention studies	Fruit and vegetable and potassium intake, but more specifically some specific types of fruit and berry juices	Vascular function	SR: Mixed results; effects on healthy population less clear. May have beneficial effects in population at high risk.	A2: Not fully evaluated.<br><br>Regarding fruit juice: Should be covered by D'Elia. Is partly covered by studies on berries, but not completely.<br><br>Regarding fruit and vegetables in general – it includes a few studies (n=3) on tomato puree and some studies on orange juice, otherwise mostly berry juice.
**RCTs**					
Body weight					
Cardiometabolic risk factors, inflammation, antioxidant status					
**Ayoub-Charette et al 2021 (158)**	11 Jan 2021<br>Feeding trials<br>n=8 for fruit juice in addition trials	Food sources of fructose	Uric acid	MA: 100% fruit juice decreased uric acid (while SSB increase uric acid), high certainty of evidence	A2: Moderate
Cowan et al 2019 (159)	2 November 2018<br>RCTs (>1w)<br>Adults BMI>25 (weight stable)<br>n=33 in total<br>n=5 for orange or grapefruit juice<br>n=1 for cherry juice	Whole foods or dietary patterns, but not fruit and vegetables per se. Six studies on juice.	Markers of subclinical inflammation	SR: Mixed results (some positive, some neutral) regarding the 6 studies on citrus and tart fruits. effects. Methodological limitations, short duration (mostly 4 or 8 weeks, 10-25 participants). Likely underpowered. Also, inflammation levels were in all studies but one near healthy at baseline.	A2: High (11 of 13)<br>However, only 6 studies on juice.<br><br>Not included because of too little and unclear evidence.
Choo et al 2018 (160)	25 April 2018<br>Controlled interventions studies<br>n=155 in total	Food sources of fructose-containing sugars. Did not search for fruit juice (or fruit) specifically.	Glycaemic control (glycated haemoglobin, fasting blood glucose, fasting blood glucose insulin)	MA: Substitution studies: fruit juice showed positive effects (decreasing blood insulin)	Not primarily focused on fruit juice, a different angle. Not mentioned by D'Elia. However, similar conclusions, i.e., no adverse effects.

					Not a study on effects of fruits or fruit juice per se (these terms are not included in their search terms).
Murphy et al 2017 (161)	14 April 2016<br>RCTs<br>All ages<br>n=18<br><br>One study on children	Fruit juice	Glucose control and insulin sensitivity	No significant association	Should be covered by D'Elia.
**Tonin et al 2015 (162)**	July 2015<br>RCTs<br>n=28, of which 16 used for meta-analyses<br><br>Includes on 5 tomato juice, 1 carrot juice, the rest fruit juice or berry juice.<br><br>4 studies after 2013 (comparison with Crow-White)	Natural fruit or vegetable juices vs placebo or their beverages	Plasma antioxidant status (different markers)	MA: Positive association for some types of fruit and vegetables juices or some two of the markers (vitamin C and reducing MDA), but not for the other.<br><br>Overall poor quality of the studies.<br><br>MA based on 16 studies, but much fewer for the different markers. See fig 2. Vit C is based on 2 studies on black current and 1 on grape. MDA is based on goji berry, cranberry, tomato, 2 pomegranates.	A2: Medium (10.5)<br><br>Weak evidence, but an indication.
Wang et al 2014 (163)	March 2014<br>RCTs<br>Adults<br>n=12	Fruit juice	Glucose control and insulin sensitivity	No significant association	Should be covered by D'Elia and Motallaei.
**Crowe-White et al 2017 (164)**	November 2013 (?! published 4 years later)<br>Mixed<br>n=10 for antioxidant status<br>n=5 for lipid profiles<br>(all were intervention studies, except for 2 cross-sectional studies)	100% fruit juice	Antioxidant status<br>Lipid profile	SR: Limited evidence suggests potential improvements on the outcomes.	A2: Low (modified NNR AMSTAR2)<br><br>Mostly RCTs and 2 cross-sectional.<br><br>Lipid profiles should be covered by D'Elia.
Liu K et al 2013 (165)	October 2012<br>RCTs<br>n=19	100% fruit juice	Serum cholesterol<br>Blood pressure	Borderline reduction of diastolic blood pressure. No effect on the other outcomes	Should be covered by D'Elia and Liu 2019.<br><br><br><br>A bit old.
**Other**					
Zheng et al 2017 (166)	Not stated<br>Not a systematic review, but a comprehensive	Vegetable and fruit juice	CVD risk factors	Lowering blood pressure, improving blood lipids.	Not completely relevant, a summary of effects of and mechanisms for fruit and

	summary of studies on fruit and vegetable juices on CVD risk factors, particularly blood lipids and blood pressure				vegetable juices, not a proper systematic review.

Not relevant

Léon et al 2022	University students	Eating behaviour	Weight gain		Fruit and vegetables not specifically investigated
Khanna et al 2019	Dec 2017<br>All ages<br>Observational studies<br>Trials	Nutrition, food	Depression		Fruit and vegetables not investigated
Guzek et al 2022	June 2019<br>Woman	Dietary patterns with fruit and vegetables.	Mental health		Dietary patterns
Godos et al 2921	Nov 2019<br>Observational studies	Diet	Sleep quality	Not relevant	Fruit and vegetables not investigated
Fogelholm et al 2012		Dietary composition, fibre, sugar	Obesity, weight change	Not relevant	Not relevant exposure
English et al 2019	Not an article	Complementary foods (breastfeeding)	Growth, body composition, ow, ob	Not relevant	Not relevant
Roulund Wilken et al 2022<br>*Found in updated search in May*	April 2020<br>Metabolic syndrom patients<br>RCTs	Anthocyanin-rich berries	Lipids, glucose, insulin blood pressure, BMR	Statistically significant results only seen for LDL, but also for TG for strawberries.	Not fully evaluated. Only on specific types of berries, not berries in general.
Wang et al 2020	June 2019<br>RCTs, adults<br>n=13	Dietary citrus and/or its extracts intake	Weight loss	Significant beneficial effect on body weight.	Study on single fruit
Driessche et al 2018	25 April 2017<br>Intervention studies<br>n=8 for blueberries<br>n=8 for cranberries<br>n=3 for goji berries<br>n=7 for strawberries	Superfoods, including fruits and vegetables and berries	Components in the metabolic syndrome	Limited evidence	Studies on single berries
Paixao et al 2019	November 2018<br>Weight control registries<br>n=	Psychological and behavioural factors	Long term weight maintenance	Increasing vegetable consumption was associated with weight	Not population-based
Noll et al 2021	August 2019<br>Postmenopausal women	Dietary intake	Postmenopausal symptoms		Fruit and vegetables not investigated
Arab et al 2019	October 2018<br>RCTs, cross-sectional, observational (?)<br>n=18 (mostly RCTs)	Diet	Mood states		Studies on diets, not single foods

Aucoin et al 2020	15 April 2018	Diet	Psychosis		A scoping review
Chapman et al 2019	August 2017<br>All types of studies<br>n=18	Diet or food intake, fruit and vegetables not included	Age-related macular degeneration		Fruit and vegetables not included as an exposure
Ghouri et al 2018	July 2017<br>Cohort, case-control, RCTs<br>n=2 for cranberry juice	Non-antibiotic measures, included cranberry juice	Urinary tract infections in pregnancy	No support of an effect.	Fruit and vegetables not included as an exposure
Zhao et al 2016	October 2015<br>n=1 cohort for fruit<br>n=3 case-control for fruit<br>n=1 cohort for vegetables<br>n=3 case-control for vegetables	Fruit, vegetables, fat and red and processed meat	Barrett's esophagus risk	Vegetables may be protective.	Precursor for oesophageal adenocarcinoma – not relevant.
Grosso et al 2017	March 2017<br>Cohort studies<br>n=37 for F&V combined<br>n=21 for fruit<br>n=24 for vegetables<br>Associations in cohorts presenting associations between diet and cancer risk	Meat, fruits, and vegetables	Health risk factors, including body weight (and also smoking, PA, education, alcohol)	No relation to BMI.	The purpose was to study clusters of factors.
Han et al 2017	March 2016<br>Epidemiologic studies (Intervention studies, RCTs excluded)<br>n=71<br>n=64 cross-sectional<br>n=6 cohort studies<br>n=1 case-control	Determinants of hyperhomocystein	Hyperhomocystein	Fruit intake protective for hyperhomocystein only seen in the original study. Not seen or investigated in the SR.	Not only a systematic review, also an original article (a population-based study). Almost entirely based on cross-sectional studies. Too broad, not focused on fruit and vegetables.
Garcia-Larsen 2018	December 2017 for intervention studies<br>13 July 2013 for observational studies<br>Children and young adults <40 yrs<br>n=35 for fruit and vegetables and nuts	Breastfeeding, maternal and infant dietary exposures<br>Dietary patterns, nutrient intake, foods	Allergic or autoimmune disease (not rare diseases): Asthma, wheezing, eczema, allergic rhinitis, food allergy		Fruit and vegetables not studied
Lai et al 2014	Aug 2013<br>Community-dwelling adults, ≥18 y old<br>Observational and interventions	Dietary patterns	Depression		Dietary patterns
Li R et al 2018	June 2017<br>Cohort and cross-sectional	Dietary factors: red meat, seafoods, alcohol, fructose, dairy	Risk of gout and hyperuricemia		Fruit and vegetables not studied

		products, soy foods, high-purine vegetables or coffee			
Li Y et al 2017	September 2016	Dietary patterns	Depression		Dietary patterns
Sanhueza et al 2012	May 2010	Some nutrients, foods and dietary patterns, not fruit and vegetables	Unipolar depression		Fruit and vegetables not studied
Turner-McGrievy et al 2014	February 2014	Vegetarian and vegan diets, and food groups associated with the identified factors	Metabolic syndrome		Fruit and vegetables not part of the systematic literature review.
Bach et al 2019	February 2019	Dietary patterns	CKD (chronic kidney disease)		Dietary patterns

HL: * The studies were first evaluated using the non-modified AMASTAR 2, for which ≥11/16 (for MA) or ≥9/13 (for SR) was considered as high (approx. 70%) and <50 % as low. At a later stage, the most relevant studies were re-evaluated using the modified AMSTAR 2.

Summary of findings in Aune (2017), Zurbau (2020), Bechthold (2017), Schwingshackl (2017) and Halvorsen (2021)

High versus low and dose-response regarding fruit and vegetables intake. All risk reductions given below are statistically significant unless otherwise indicated.

The confidence intervals get broader at higher intakes (fewer studies).

Links to figures:
Aune 2017: https://pubmed.ncbi.nlm.nih.gov/28338764/ (fig 2-6)
Zurbau 2021: https://www.ncbi.nlm.nih.gov/pmc/articles/PMC7792377/ (suppl pdf, fig S33-S35, S44-S46, S90-S92, S102-S104, S147-S149, S158-S160)
Bechthold 2017: https://www.tandfonline.com/doi/full/10.1080/10408398.2017.1392288 (fig 3, fig 4)
Schwingshackl 2017: https://academic.oup.com/ajcn/article/105/6/1462/4569801 (fig 2)

	Total fruit and vegetables intake	Fruit intake	Vegetables intake
Aune 2017 (13)			
CHD (incidence and mortality)	High versus low<br>13 % risk reduction.<br>Dose-response<br>Almost linear risk reduction up to 800 g/d.<br>Around 25 % risk reduction at 800 g/d.	High versus low<br>14 % risk reduction.<br>Dose-response<br>Steeper risk reductions up to 200 g/d.	High versus low<br>13 % risk reduction.<br>Dose-response<br>Steeper risk reductions up to 200-300 g/d.
Stroke (incidence and mortality)	High versus low<br>21 % risk reduction.<br>Dose-response<br>Steeper risk reductions up to 400 g/d.<br>Around 25 % risk reduction at 400 g/d.<br>Around 30 % risk reduction at 800 g/d.	High versus low<br>18 % risk reduction.<br>Dose-response<br>Risk reductions up to 200 g/d, after that the curve goes slightly up again.	High versus low<br>13 % risk reduction.<br>Dose-response<br>Steeper risk reductions up to 200-300 g/d.
CVD (incidence and mortality)	High versus low<br>16 % risk reduction.<br>Dose-response<br>Steeper risk reductions up to 400-500 g/d, but continued risk reductions.<br>Around 20 % risk reduction at 400 g/d.<br>Around 26-27 % risk reduction at 800 g/d.	High versus low<br>13 % risk reduction.<br>Dose-response<br>Steeper risk reductions up to 200-300 g/d.	High versus low<br>11 % risk reduction.<br>Dose-response<br>Almost linear risk reductions up to 600 g/d.
Total cancer (incidence and mortality) Not relevant, covered by a qSR.	High versus low<br>7 % risk reduction.<br>Dose-response<br>Steeper risk reductions up to 400 g/d.<br>Around 10 % risk reduction at 400 g/d.<br>Small change at 800 g/d.	High versus low<br>7 % risk reduction.<br>Dose-response<br>Smaller reductions, almost linear.	High versus low<br>5 % risk reduction.<br>Dose-response<br>Smaller reductions.
All-cause mortality	High versus low<br>18 % risk reduction.<br>Dose-response<br>Steeper risk reductions up to 400 (or 400-500) g/d, but continued risk reductions up to 800 g/d.<br>Around 24 % risk reduction at 400 g/d.	High versus low<br>13 % risk reduction.<br>Dose-response<br>Risk reductions up to 200 g/d	High versus low<br>13 % risk reduction.<br>Dose-response<br>Risk reductions up to 200-300 g/d, then small additional reductions.

	Around 30 % risk reduction at 800 g/d.		
Zurbau 2020 (11)			
CVD incidence	High versus low<br>7 % risk reduction.<br>Dose-response<br>Continuous risk reductions up to 10 servings/d.<br>Around 20 % risk reduction at the highest intake.	High versus low<br>9 % risk reduction.<br>Dose-response<br>Continuous risk reductions up to 10 servings/day.	High versus low<br>6 % risk reduction.<br>Dose-response<br>Continuous risk reductions up to 10 servings/day
CVD mortality	High versus low<br>11 % risk reduction.<br>Dose-response<br>Steeper risk reductions up to 4 servings/d, and after 5 servings no change<br>Around 20 % risk reduction at 4 servings.	High versus low<br>12 % risk reduction.<br>Dose-response<br>Steeper risk reductions up to 2-3 servings/d, and additional risk reductions up to 7 servings	High versus low<br>13 % risk reduction.<br>Dose-response<br>Risk reductions up to 2-3 servings/d
CHD incidence	High versus low<br>12 % risk reduction.<br>Dose-response<br>Risk reductions up to 4 servings/d<br>Around 15 % risk reduction at 4 servings/d,	High versus low<br>12 % risk reduction.<br>Dose-response<br>Risk reductions up to 2 servings/day	High versus low<br>8 % risk reduction.<br>Dose-response<br>Continuous risk reductions up to 7 servings/d.<br>Nearly 20 % risk reduction at the highest intake.
CHD mortality	High versus low<br>19 % risk reduction.<br>Dose-response<br>Steeper risk reductions up to 3 servings/d<br>Around 30 % risk reduction at 3 servings/d,	High versus low<br>14 % risk reduction.<br>Dose-response<br>Steeper risk reductions up to 2 servings/d	High versus low<br>14 % risk reduction.<br>Dose-response<br>Continuous small risk reductions up to 4 servings/d.
Stroke incidence	High versus low<br>18 % risk reduction.<br>Dose-response<br>Slightly steeper risk reductions up to 4 servings/d, but continuous risk reductions up to 9 servings/d.<br>Around 20 % risk reduction at 4 servings/d,<br>Around 30 % risk reduction at 9 servings/d.	High versus low<br>18 % risk reduction.<br>Dose-response<br>Risk reductions up to 2 servings/d	High versus low<br>12 % risk reduction.<br>Dose-response<br>Risk reductions up to 1-2 servings/d
Stroke mortality	High versus low<br>27 % risk reduction.<br>Dose-response<br>Continuous risk reductions up to 10 servings/d<br>Around 30 % risk reduction at the highest intake (spline model).	High versus low<br>13 % risk reduction.<br>Dose-response<br>Risk reductions up to 2 servings/d	High versus low<br>6 % risk reduction.<br>Dose-response<br>Risk reductions up to 2-3 servings/d
Bechthold 2017 (12)			
CHD	-	High versus low<br>8 % risk reduction.<br>Dose-response<br>Risk reductions up to 200 g/d.	High versus low<br>11 % risk reduction.<br>Dose-response<br>Steeper risk reductions up to 200-300 g/d.<br>Continued risk reduction up to 600 g/d.
Stroke	-	High versus low<br>13 % risk reduction.	High versus low<br>17 % risk reduction.

		Dose-response<br>Risk reductions up to 200 g/d.	Dose-response<br>Risk reductions up to 200 g/d.
Heart failure	-	High versus low<br>No risk reduction.	High versus low<br>5 % risk reduction.
Schwingschackl 2017 (21)			
All-cause mortality	-	High versus low<br>7 % risk reduction.<br>Dose-response<br>Risk reductions up to 200 g/d.	High versus low<br>9 % risk reduction.<br>Dose-response<br>Steeper risk reductions up to 200-300 g/d.
Halvorsen 2021 (23)			
T2D risk	High versus low<br>7 % risk reduction.<br>Dose-response<br>9-10 % risk reductions at 600-700 g/d, borderline significant	High versus low<br>7 % risk reduction<br>Dose-response<br>8-12 % risk reductions at 100-500 g/d.	High versus low<br>5 % risk reduction (CI 0.88-1.02).<br>Dose-response<br>12-14 % risk reductions at 200-400 g/d

Findings regarding specific types of fruit and vegetables

Similar significant findings are indicated in blue.

	Zurbau						Aune					Halvorsen
	CVD incidence	CHD incidence	Stroke incidence	CVD mortality	CHD mortality	Stroke mortality	CHD	Stroke (total)	CVD	All-cause mortality	Cancer	T2D
Apple/pears (pommes)	0.76 (L)	0.90 (VL)	0.89 (M)	0.86 (VL)	0.84 (VL)	NS	0.85	0.88	0.86	0.80	-	0.79/0.88*
Apricots	-	-	-	NS	-	-						
Bananas	-	-	-	NS	NS	NS	NS	-	-	NS	-	NS
Berries	NS	NS	NS	NS	NS	NS	NS	NS	NS	0.92	-	NS
Blueberries												0.76
Citrus fruit	0.88 (L)	0.91 (L)	0.88 (L)	NS	0.91 (VL)	0.90 (L)	0.91	0.74	0.78	0.90	NS	NS
Citrus fruit juice							-	NS	NS	-	-	-
Fruit juice	NS	NS	0.82 (VL)	NS	BP	0.67 (L)	0.79	0.67	NS	0.87	NS	NS
Dried fruit	-	-	-	NS	NS	NS	NS	NS	NS	-	NS	-
Grapes	-	NS	-	BP	NS	BP	NS	NS	NS	-	-	0.83**
Strawberries							NS	-	NS	-	-	NS
Watermelon	-	NS	-	-	-	-	NS	NS	-	-	-	NS
Cantaloup												1.11
Oranges												NS
Prunes												NS
Peaches, plums, apricot												NS
Tinned fruits							-	-	1.23	1.13	NS	-
Pickled vegetables							-	0.80	-	-	NS	-
Allium	NS	NS	0.89 (VL)	0.33 (VL)	0.67 (VL)	NS	NS	BP	-	NS	-	NS
Onions							BP	-	-	NS	NS	-

Broccoli							-	-	NS	-	NS	NS
Carrots	-	-	-	NS	NS	0.54 (L)	-	-	0.81	-	-	-
Cruciferous	NS	NS	NS	0.85 (VL)	0.91 (VL)	BP	NS	NS	NS/BP	0.88	0.84	NS
Green leafy	0.87 (L)	0.82 (M)	0.88 (L)	0.87 (L)	0.86 (VL)	0.90 (L)	0.83	0.88	0.84	0.92	-	BP
Noncruciferous							-	-	0.76	-	NS	-
Potatoes							NS	NS	NS	0.78	NS	NS***
Root vegetables								NS	-	NS	-	-
Raw vegetables							NS	-	NS/BP	0.88	-	-
Cabbage												1.10
Brussel sprouts												1.10
Kale, mustard, chard												1.18
Celery	-	-	-	NS	NS	-						
Tomatoes	NS	NS	0.20 (L)	NS	NS	NS	BP	NS	NS	-	0.86	NS
Beta-carotene rich							0.83	-	NS	-	-	
Lutein rich							NS	-	NS	-	-	
Lycopene rich							NS	-	-	-	-	
Vitamin C rich							0.86	-	-	-	-	
Cooked vegetables							-	-	-	0.87	-	
Green vegetables							-	-	-	-	NS	
Green yellow vegetables							-	-	-	-	BP	
Yellow vegetables							-	-	-	-	NS	BP
Salads							-	-	-	-	NS	

BP = Borderline protective
NS = Non-significant association
M = Moderate level of evidence (according to GRADE)
L = Low level of evidence (according to GRADE)
VL = Very low level of evidence (according to GRADE)
*Apples and apples/pears, ** Grapes and raisins, *** significant positive dose-response

Adjustments made in the observational studies in the meta-analyses/systematic reviews included in the summary.

Outcome	Meta-analysis or systematic review	Adjustments	Comments in the article
Cancer	The CUP-report (WCRF/AICR)	Details are not available. Should be similar to other large cohort studies below, seen in e.g., Aune.	
CVD	Aune 2017	Most of studies have adjusted for age, sex, smoking, BMI. Many have also adjusted for social class, physical activity, energy intake and other factors.	Authors conclusion: Associations appear to be independent of adiposity.
	Zurbau 2020	Same as above.	
	Bechthold 2017	Same as above.	
All-cause mortality	Aune 2017	See above.	
	Schwingschackl 2017	Same as above.	
T2D	Halvorsen 2021	Same as above.	Most studies adjusted for baseline BMI. Few studies conducted analyses with and without adjustments for BMI to assess potential mediation. No studies tested whether adjustment for weight change during follow-up, which could also mediate part of the inverse association.
Bone health	Brondani 2019	Almost all cohort studies (n=6) have adjusted for age, gender, BMI, smoking, vitamin D and calcium supplements. Some have also adjusted for education.	
	Hu 2018	Most studies have adjusted for age, BMI, smoking. Many also adjusted for other factors such as hormone replacement, other common risk factors of PMOP.	
Mental health	Matison 2021	Most studies have adjusted for smoking, alcohol, BMI and other factors.	
	Saghafian 2018	Most studies have adjusted for age, education, BMI, smoking energy intake.	
	Dharmyani 2021	SR: Key confounders are adjusted for, not clearly described which.	
Cognitive function	Wu 2017	Almost all studies have adjusted for age, sex and education. Some also adjusted for BMI. Other factors are also adjusted for.	
Frailty	Granic 2020	Info is lacking, but RoB is clearly discussed.	
	Ghoreishy 2021	Most studies have adjusted for age, sex and BMI. Many other factors, including education, are also adjusted for.	
Gallstone disease	Zhang 2019	Most studies have adjusted for age, sex, education, smoking, BMI	
IBD	Li 2015	Most studies have adjusted for age and sex. Some also for BMI and education. Unknown for some studies.	
	Milajerdi 2020	Most studies have adjusted for age and sex and smoking. Some also for BMI, education. Other factors are also adjusted for.	
Periodontal disease	O'Conner 2020	SR: Most studies have adjusted for sex, smoking, BMI, education, and other factors.	
Eye disease	Huang 2015	Most studies have adjusted for age, sex, smoking, BMI/obesity, education. Other factors are also adjusted for.	

	Dinu 2019	Most studies have adjusted for age, sex, smoking, BMI/obesity or total energy intake, education. Other factors are also adjusted for.	
Wheezing and asthma	Seyedrezazadeh 2015	Most studies have adjusted for age, smoking, education. Some also for birth weight/overweight/BMI. Other factors are also adjusted for.	
Hypertension	Schwingschackl 2017	Most studies have adjusted for age, smoking, BMI, and many other factors.	
	Wu 2016	Same as above.	
Body weight	Schlesinger 2019	Most studies have adjusted for age, smoking, physical activity, other dietary factors, BMI or body weight at baseline and other factors.	
	Nour 2018	Same as above.	
Hypertension	Schwingschackl 2017	Most studies have adjusted for age, smoking, BMI, physical activity, and many other factors.	
	Wu 2016	Same as above.	
Metabolic syndrome	Lee 2019	Most studies have adjusted for age, sex, smoking, physical activity, and other factors. Some studies also for energy intake and BMI.	
	Zhang & Zhang 2017	Most studies have adjusted for age, sex, and other factors. Some also for energy intake and BMI.	

Abbreviations;
* SR: systematic review(s); MA: meta-analysis, RCTs: randomized controlled trials; CVD: cardiovascular disease; CHD: coronary heart disease; T2D: type 2 diabetes.

LIST OF REFERENCES

1. Wang Y, Lu J, Wen N, Nie G, Peng D, Xiong X, et al. The role of diet and nutrition related indicators in biliary diseases: an umbrella review of systematic review and meta-analysis. Nutr Metab (Lond). 2022;19(1):51.
2. Webster J, Rycroft CE, Greenwood DC, Cade JE. Dietary risk factors for hip fracture in adults: An umbrella review of meta-analyses of prospective cohort studies. PLoS One. 2021;16(11):e0259144.
3. Wallace TC, Bailey RL, Blumberg JB, Burton-Freeman B, Chen CO, Crowe-White KM, et al. Fruits, vegetables, and health: A comprehensive narrative, umbrella review of the science and recommendations for enhanced public policy to improve intake. Crit Rev Food Sci Nutr. 2020;60(13):2174-211.
4. Kwok CS, Gulati M, Michos ED, Potts J, Wu P, Watson L, et al. Dietary components and risk of cardiovascular disease and all-cause mortality: a review of evidence from meta-analyses. Eur J Prev Cardiol. 2019;26(13):1415-29.
5. Yip CSC, Chan W, Fielding R. The Associations of Fruit and Vegetable Intakes with Burden of Diseases: A Systematic Review of Meta-Analyses. J Acad Nutr Diet. 2019;119(3):464-81.
6. Angelino D, Godos J, Ghelfi F, Tieri M, Titta L, Lafranconi A, et al. Fruit and vegetable consumption and health outcomes: an umbrella review of observational studies. Int J Food Sci Nutr. 2019;70(6):652-67.
7. Micha R, Shulkin ML, Peñalvo JL, Khatibzadeh S, Singh GM, Rao M, et al. Etiologic effects and optimal intakes of foods and nutrients for risk of cardiovascular diseases and diabetes: Systematic reviews and meta-analyses from the Nutrition and Chronic Diseases Expert Group (NutriCoDE). PLoS One. 2017;12(4):e0175149.
8. Deng C, Lu Q, Gong B, Li L, Chang L, Fu L, et al. Stroke and food groups: an overview of systematic reviews and meta-analyses. Public Health Nutr. 2018;21(4):766-76.
9. Sun T, Zhang Y, Ding L, Li T, Li Q. The Relationship Between Major Food Sources of Fructose and Cardiovascular Outcomes: A Systematic Review and Dose-Response Meta-Analysis of Prospective Studies. Adv Nutr. 2022.
10. Bhandari B, Liu Z, Lin S, Macniven R, Akombi-Inyang B, Hall J, et al. Long-Term Consumption of 10 Food Groups and Cardiovascular Mortality: A Systematic Review and Dose Response Meta-Analysis of Prospective Cohort Studies. Adv Nutr. 2023;14(1):55-63.
11. Zurbau A, Au-Yeung F, Blanco Mejia S, Khan TA, Vuksan V, Jovanovski E, et al. Relation of Different Fruit and Vegetable Sources With Incident Cardiovascular Outcomes: A Systematic Review and Meta-Analysis of Prospective Cohort Studies. J Am Heart Assoc. 2020;9(19):e017728.
12. Bechthold A, Boeing H, Schwedhelm C, Hoffmann G, Knüppel S, Iqbal K, et al. Food groups and risk of coronary heart disease, stroke and heart failure: A systematic review and dose-response meta-analysis of prospective studies. Crit Rev Food Sci Nutr. 2019;59(7):1071-90.
13. Aune D, Giovannucci E, Boffetta P, Fadnes LT, Keum N, Norat T, et al. Fruit and vegetable intake and the risk of cardiovascular disease, total cancer and all-cause mortality-a systematic review and dose-response meta-analysis of prospective studies. Int J Epidemiol. 2017;46(3):1029-56.
14. Zhan J, Liu YJ, Cai LB, Xu FR, Xie T, He QQ. Fruit and vegetable consumption and risk of cardiovascular disease: A meta-analysis of prospective cohort studies. Crit Rev Food Sci Nutr. 2017;57(8):1650-63.
15. Lippi G, Mattiuzzi C, Franchini M. Vegetables intake and venous thromboembolism: a systematic review. Blood Coagul Fibrinolysis. 2016;27(3):242-5.
16. Gan Y, Tong X, Li L, Cao S, Yin X, Gao C, et al. Consumption of fruit and vegetable and risk of coronary heart disease: a meta-analysis of prospective cohort studies. Int J Cardiol. 2015;183:129-37.
17. Hu D, Huang J, Wang Y, Zhang D, Qu Y. Fruits and vegetables consumption and risk of stroke: a meta-analysis of prospective cohort studies. Stroke. 2014;45(6):1613-9.
18. Wang X, Ouyang Y, Liu J, Zhu M, Zhao G, Bao W, et al. Fruit and vegetable consumption and mortality from all causes, cardiovascular disease, and cancer: systematic review and dose-response meta-analysis of prospective cohort studies. BMJ. 2014;349:g4490.
19. Sherzai A, Heim LT, Boothby C, Sherzai AD. Stroke, food groups, and dietary patterns: a systematic review. Nutr Rev. 2012;70(8):423-35.

20. Wang DD, Li Y, Bhupathiraju SN, Rosner BA, Sun Q, Giovannucci EL, et al. Fruit and Vegetable Intake and Mortality: Results From 2 Prospective Cohort Studies of US Men and Women and a Meta-Analysis of 26 Cohort Studies. Circulation. 2021;143(17):1642-54.
21. Schwingshackl L, Schwedhelm C, Hoffmann G, Lampousi AM, Knüppel S, Iqbal K, et al. Food groups and risk of all-cause mortality: a systematic review and meta-analysis of prospective studies. Am J Clin Nutr. 2017;105(6):1462-73.
22. Aasheim ET, Sharp SJ, Appleby PN, Shipley MJ, Lentjes MA, Khaw KT, et al. Tinned fruit consumption and mortality in three prospective cohorts. PLoS One. 2015;10(2):e0117796.
23. Halvorsen RE, Elvevstad M, Molin M, Aune D. Fruit and vegetable consumption and the risk of type 2 diabetes: a systematic review and dose-response meta-analysis of prospective studies. BMJ NPH. 2021;bmjnph-2020-000218. doi: 10.1136/bmjnph-2020-000218.
24. Chen GC, Koh WP, Yuan JM, Qin LQ, van Dam RM. Green leafy and cruciferous vegetable consumption and risk of type 2 diabetes: results from the Singapore Chinese Health Study and meta-analysis. Br J Nutr. 2018;119(9):1057-67.
25. Schwingshackl L, Hoffmann G, Lampousi AM, Knüppel S, Iqbal K, Schwedhelm C, et al. Food groups and risk of type 2 diabetes mellitus: a systematic review and meta-analysis of prospective studies. Eur J Epidemiol. 2017;32(5):363-75.
26. Jia X, Zhong L, Song Y, Hu Y, Wang G, Sun S. Consumption of citrus and cruciferous vegetables with incident type 2 diabetes mellitus based on a meta-analysis of prospective study. Prim Care Diabetes. 2016;10(4):272-80.
27. Wang PY, Fang JC, Gao ZH, Zhang C, Xie SY. Higher intake of fruits, vegetables or their fiber reduces the risk of type 2 diabetes: A meta-analysis. J Diabetes Investig. 2016;7(1):56-69.
28. Wu Y, Zhang D, Jiang X, Jiang W. Fruit and vegetable consumption and risk of type 2 diabetes mellitus: a dose-response meta-analysis of prospective cohort studies. Nutr Metab Cardiovasc Dis. 2015;25(2):140-7.
29. Li M, Fan Y, Zhang X, Hou W, Tang Z. Fruit and vegetable intake and risk of type 2 diabetes mellitus: meta-analysis of prospective cohort studies. BMJ Open. 2014;4(11):e005497.
30. Li S, Miao S, Huang Y, Liu Z, Tian H, Yin X, et al. Fruit intake decreases risk of incident type 2 diabetes: an updated meta-analysis. Endocrine. 2015;48(2):454-60.
31. Muraki I, Imamura F, Manson JE, Hu FB, Willett WC, van Dam RM, et al. Fruit consumption and risk of type 2 diabetes: results from three prospective longitudinal cohort studies. BMJ. 2013;347:f5001.
32. Cooper AJ, Forouhi NG, Ye Z, Buijsse B, Arriola L, Balkau B, et al. Fruit and vegetable intake and type 2 diabetes: EPIC-InterAct prospective study and meta-analysis. Eur J Clin Nutr. 2012;66(10):1082-92.
33. Machairiotis N, Vasilakaki S, Minns L, Malakasis A. Nutrients that modulate gestational diabetes mellitus: A systematic review of cohort studies Jan 2019-Jan 2020. Int J Clin Pract. 2021;75(8):e14033.
34. Mohammadi T, Irandoost P, Roshanravan N, Khamechi SP, Milajerdi A, Larijani B, et al. Is there any association between fruit consumption and the risk of gestational diabetes mellitus? A systematic review and meta-analysis. Complement Ther Med. 2020;54:102445.
35. Mijatovic-Vukas J, Capling L, Cheng S, Stamatakis E, Louie J, Cheung NW, et al. Associations of Diet and Physical Activity with Risk for Gestational Diabetes Mellitus: A Systematic Review and Meta-Analysis. Nutrients. 2018;10(6).
36. Schoenaker DA, Mishra GD, Callaway LK, Soedamah-Muthu SS. The Role of Energy, Nutrients, Foods, and Dietary Patterns in the Development of Gestational Diabetes Mellitus: A Systematic Review of Observational Studies. Diabetes Care. 2016;39(1):16-23.
37. Schwingshackl L, Schwedhelm C, Hoffmann G, Knüppel S, Iqbal K, Andriolo V, et al. Food Groups and Risk of Hypertension: A Systematic Review and Dose-Response Meta-Analysis of Prospective Studies. Adv Nutr. 2017;8(6):793-803.
38. Wu L, Sun D, He Y. Fruit and vegetables consumption and incident hypertension: dose-response meta-analysis of prospective cohort studies. J Hum Hypertens. 2016;30(10):573-80.
39. Li B, Li F, Wang L, Zhang D. Fruit and Vegetables Consumption and Risk of Hypertension: A Meta-Analysis. J Clin Hypertens (Greenwich). 2016;18(5):468-76.

40. Kalyoncu ZB, Pars H, Bora-Güneş N, Karabulut E, Aslan D. A systematic review of nutrition-based practices in prevention of hypertension among healthy youth. Turk J Pediatr. 2014;56(4):335-46.
41. Collese TS, Nascimento-Ferreira MV, de Moraes ACF, Rendo-Urteaga T, Bel-Serrat S, Moreno LA, et al. Role of fruits and vegetables in adolescent cardiovascular health: a systematic review. Nutr Rev. 2017;75(5):339-49.
42. Adegbola A, Behrendt CA, Zyriax BC, Windler E, Kreutzburg T. The impact of nutrition on the development and progression of peripheral artery disease: A systematic review. Clin Nutr. 2022;41(1):49-70.
43. Hosseini B, Berthon BS, Saedisomeolia A, Starkey MR, Collison A, Wark PAB, et al. Effects of fruit and vegetable consumption on inflammatory biomarkers and immune cell populations: a systematic literature review and meta-analysis. Am J Clin Nutr. 2018;108(1):136-55.
44. Kodama S, Horikawa C, Fujihara K, Ishii D, Hatta M, Takeda Y, et al. Relationship between intake of fruit separately from vegetables and triglycerides - A meta-analysis. Clin Nutr ESPEN. 2018;27:53-8.
45. Lee DPS, Low JHM, Chen JR, Zimmermann D, Actis-Goretta L, Kim JE. The Influence of Different Foods and Food Ingredients on Acute Postprandial Triglyceride Response: A Systematic Literature Review and Meta-Analysis of Randomized Controlled Trials. Adv Nutr. 2020;11(6):1529-43.
46. Hartley L, Igbinedion E, Holmes J, Flowers N, Thorogood M, Clarke A, et al. Increased consumption of fruit and vegetables for the primary prevention of cardiovascular diseases. Cochrane Database Syst Rev. 2013(6):CD009874.
47. Schwingshackl L, Hoffmann G, Iqbal K, Schwedhelm C, Boeing H. Food groups and intermediate disease markers: a systematic review and network meta-analysis of randomized trials. Am J Clin Nutr. 2018;108(3):576-86.
48. Lee M, Lim M, Kim J. Fruit and vegetable consumption and the metabolic syndrome: a systematic review and dose-response meta-analysis. Br J Nutr. 2019;122(7):723-33.
49. Zhang Y, Zhang DZ. Associations of vegetable and fruit consumption with metabolic syndrome. A meta-analysis of observational studies. Public Health Nutr. 2018;21(9):1693-703.
50. Tian Y, Su L, Wang J, Duan X, Jiang X. Fruit and vegetable consumption and risk of the metabolic syndrome: a meta-analysis. Public Health Nutr. 2018;21(4):756-65.
51. Shin JY, Kim JY, Kang HT, Han KH, Shim JY. Effect of fruits and vegetables on metabolic syndrome: a systematic review and meta-analysis of randomized controlled trials. Int J Food Sci Nutr. 2015;66(4):416-25.
52. Poorolajal J, Sahraei F, Mohamdadi Y, Doosti-Irani A, Moradi L. Behavioral factors influencing childhood obesity: a systematic review and meta-analysis. Obes Res Clin Pract. 2020.
53. Schlesinger S, Neuenschwander M, Schwedhelm C, Hoffmann G, Bechthold A, Boeing H, et al. Food Groups and Risk of Overweight, Obesity, and Weight Gain: A Systematic Review and Dose-Response Meta-Analysis of Prospective Studies. Adv Nutr. 2019;10(2):205-18.
54. Nour M, Lutze SA, Grech A, Allman-Farinelli M. The Relationship between Vegetable Intake and Weight Outcomes: A Systematic Review of Cohort Studies. Nutrients. 2018;10(11).
55. Schwingshackl L, Hoffmann G, Kalle-Uhlmann T, Arregui M, Buijsse B, Boeing H. Fruit and Vegetable Consumption and Changes in Anthropometric Variables in Adult Populations: A Systematic Review and Meta-Analysis of Prospective Cohort Studies. PLoS One. 2015;10(10):e0140846.
56. Ledoux TA, Hingle MD, Baranowski T. Relationship of fruit and vegetable intake with adiposity: a systematic review. Obes Rev. 2011;12(5):e143-50.
57. Guyenet SJ. Impact of Whole, Fresh Fruit Consumption on Energy Intake and Adiposity: A Systematic Review. Front Nutr. 2019;6:66.
58. Hebden L, O'Leary F, Rangan A, Singgih Lie E, Hirani V, Allman-Farinelli M. Fruit consumption and adiposity status in adults: A systematic review of current evidence. Crit Rev Food Sci Nutr. 2017;57(12):2526-40.
59. Mytton OT, Nnoaham K, Eyles H, Scarborough P, Ni Mhurchu C. Erratum to: systematic review and meta-analysis of the effect of increased vegetable and fruit consumption on body weight and energy intake. BMC Public Health. 2017;17(1):662.

60. Kaiser KA, Brown AW, Bohan Brown MM, Shikany JM, Mattes RD, Allison DB. Increased fruit and vegetable intake has no discernible effect on weight loss: a systematic review and meta-analysis. Am J Clin Nutr. 2014;100(2):567-76.
61. Tapsell LC, Dunning A, Warensjo E, Lyons-Wall P, Dehlsen K. Effects of vegetable consumption on weight loss: a review of the evidence with implications for design of randomized controlled trials. Crit Rev Food Sci Nutr. 2014;54(12):1529-38.
62. Brondani JE, Comim FV, Flores LM, Martini LA, Premaor MO. Fruit and vegetable intake and bones: A systematic review and meta-analysis. PLoS One. 2019;14(5):e0217223.
63. Hu D, Cheng L, Jiang W. Fruit and vegetable consumption and the risk of postmenopausal osteoporosis: a meta-analysis of observational studies. Food Funct. 2018;9(5):2607-16.
64. Benetou V, Orfanos P, Feskanich D, Michaëlsson K, Pettersson-Kymmer U, Eriksson S, et al. Fruit and Vegetable Intake and Hip Fracture Incidence in Older Men and Women: The CHANCES Project. J Bone Miner Res. 2016;31(9):1743-52.
65. Luo S, Li Y, Luo H, Yin X, Lin dR, Zhao K, et al. Increased intake of vegetables, but not fruits, may be associated with reduced risk of hip fracture: A meta-analysis. Sci Rep. 2016;6:19783.
66. Hamidi M, Boucher BA, Cheung AM, Beyene J, Shah PS. Fruit and vegetable intake and bone health in women aged 45 years and over: a systematic review. Osteoporos Int. 2011;22(6):1681-93.
67. Nowson CA, Service C, Appleton J, Grieger JA. The Impact of Dietary Factors on Indices of Chronic Disease in Older People: A Systematic Review. J Nutr Health Aging. 2018;22(2):282-96.
68. Mottaghi T, Amirabdollahian F, Haghighatdoost F. Fruit and vegetable intake and cognitive impairment: a systematic review and meta-analysis of observational studies. Eur J Clin Nutr. 2018;72(10):1336-44.
69. Wu L, Sun D, Tan Y. Intake of Fruit and Vegetables and the Incident Risk of Cognitive Disorders: A Systematic Review and Meta-Analysis of Cohort Studies. J Nutr Health Aging. 2017;21(10):1284-90.
70. Cao L, Tan L, Wang HF, Jiang T, Zhu XC, Lu H, et al. Dietary Patterns and Risk of Dementia: a Systematic Review and Meta-Analysis of Cohort Studies. Mol Neurobiol. 2016;53(9):6144-54.
71. Lamport DJ, Saunders C, Butler LT, Spencer JP. Fruits, vegetables, 100% juices, and cognitive function. Nutr Rev. 2014;72(12):774-89.
72. Loef M, Walach H. Fruit, vegetables and prevention of cognitive decline or dementia: a systematic review of cohort studies. J Nutr Health Aging. 2012;16(7):626-30.
73. Cohen JF, Gorski MT, Gruber SA, Kurdziel LB, Rimm EB. The effect of healthy dietary consumption on executive cognitive functioning in children and adolescents: a systematic review. Br J Nutr. 2016;116(6):989-1000.
74. Wang Y, Liu J, Compher C, Kral TVE. Associations between dietary intake, diet quality and depressive symptoms in youth: A systematic review of observational studies. Health Promot Perspect. 2022;12(3):249-65.
75. Matison AP, Mather KA, Flood VM, Reppermund S. Associations between nutrition and the incidence of depression in middle-aged and older adults: A systematic review and meta-analysis of prospective observational population-based studies. Ageing Res Rev. 2021;70:101403.
76. Dabravolskaj J, Marozoff S, Maximova K, Campbell S, Veugelers PJ. Relationship Between Fruit and Vegetables Intake and Common Mental Disorders in Youth: A Systematic Review. Public Health Rev. 43. Switzerland: Copyright © 2022 Dabravolskaj, Marozoff, Maximova, Campbell and Veugelers.; 2022. p. 1604686.
77. Dharmayani PNA, Juergens M, Allman-Farinelli M, Mihrshahi S. Association between Fruit and Vegetable Consumption and Depression Symptoms in Young People and Adults Aged 15-45: A Systematic Review of Cohort Studies. Int J Environ Res Public Health. 2021;18(2).
78. Głąbska D, Guzek D, Groele B, Gutkowska K. Fruit and Vegetable Intake and Mental Health in Adults: A Systematic Review. Nutrients. 2020;12(1).
79. Głąbska D, Guzek D, Groele B, Gutkowska K. Fruit and vegetables intake in adolescents and mental health: a systematic review. Rocz Panstw Zakl Hig. 2020;71(1):15-25.

80. Guzek D, Głąbska D, Groele B, Gutkowska K. Role of fruit and vegetables for the mental health of children: a systematic review. Rocz Panstw Zakl Hig. 2020;71(1):5-13.
81. Tuck NJ, Farrow C, Thomas JM. Assessing the effects of vegetable consumption on the psychological health of healthy adults: a systematic review of prospective research. Am J Clin Nutr. 2019;110(1):196-211.
82. Saghafian F, Malmir H, Saneei P, Milajerdi A, Larijani B, Esmaillzadeh A. Fruit and vegetable consumption and risk of depression: accumulative evidence from an updated systematic review and meta-analysis of epidemiological studies. Br J Nutr. 2018;119(10):1087-101.
83. Molendijk M, Molero P, Ortuño Sánchez-Pedreño F, Van der Does W, Angel Martínez-González M. Diet quality and depression risk: A systematic review and dose-response meta-analysis of prospective studies. J Affect Disord. 2018;226:346-54.
84. Liu X, Yan Y, Li F, Zhang D. Fruit and vegetable consumption and the risk of depression: A meta-analysis. Nutrition. 2016;32(3):296-302.
85. Hosseini B, Berthon BS, Wark P, Wood LG. Effects of Fruit and Vegetable Consumption on Risk of Asthma, Wheezing and Immune Responses: A Systematic Review and Meta-Analysis. Nutrients. 2017;9(4).
86. Seyedrezazadeh E, Moghaddam MP, Ansarin K, Vafa MR, Sharma S, Kolahdooz F. Fruit and vegetable intake and risk of wheezing and asthma: a systematic review and meta-analysis. Nutr Rev. 2014;72(7):411-28.
87. Nurmatov U, Devereux G, Sheikh A. Nutrients and foods for the primary prevention of asthma and allergy: systematic review and meta-analysis. J Allergy Clin Immunol. 2011;127(3):724-33.e1-30.
88. Garcia-Larsen V, Del Giacco SR, Moreira A, Bonini M, Charles D, Reeves T, et al. Asthma and dietary intake: an overview of systematic reviews. Allergy. 2016;71(4):433-42.
89. Dinu M, Pagliai G, Casini A, Sofi F. Food groups and risk of age-related macular degeneration: a systematic review with meta-analysis. Eur J Nutr. 2019;58(5):2123-43.
90. Dow C, Mancini F, Rajaobelina K, Boutron-Ruault MC, Balkau B, Bonnet F, et al. Diet and risk of diabetic retinopathy: a systematic review. Eur J Epidemiol. 2018;33(2):141-56.
91. Huang G, Wu L, Qiu L, Lai J, Huang Z, Liao L. Association between vegetables consumption and the risk of age-related cataract: a meta-analysis. Int J Clin Exp Med. 2015;8(10):18455-61.
92. O'Connor JP, Milledge KL, O'Leary F, Cumming R, Eberhard J, Hirani V. Poor dietary intake of nutrients and food groups are associated with increased risk of periodontal disease among community-dwelling older adults: a systematic literature review. Nutr Rev. 2020;78(2):175-88.
93. Skoczek-Rubińska A, Bajerska J, Menclewicz K. Effects of fruit and vegetables intake in periodontal diseases: A systematic review. Dent Med Probl. 2018;55(4):431-9.
94. Milajerdi A, Ebrahimi-Daryani N, Dieleman LA, Larijani B, Esmaillzadeh A. Association of Dietary Fiber, Fruit, and Vegetable Consumption with Risk of Inflammatory Bowel Disease: A Systematic Review and Meta-Analysis. Adv Nutr. 2021;12(3):735-43.
95. Li F, Liu X, Wang W, Zhang D. Consumption of vegetables and fruit and the risk of inflammatory bowel disease: a meta-analysis. Eur J Gastroenterol Hepatol. 2015;27(6):623-30.
96. Hou JK, Abraham B, El-Serag H. Dietary intake and risk of developing inflammatory bowel disease: a systematic review of the literature. Am J Gastroenterol. 2011;106(4):563-73.
97. Ghoreishy SM, Asoudeh F, Jayedi A, Mohammadi H. Fruit and vegetable intake and risk of frailty: A systematic review and dose response meta-analysis. Ageing Res Rev. 2021;71:101460.
98. Kojima G, Taniguchi Y, Urano T. Fruit and Vegetable Consumption and Incident Frailty in Older Adults: A Systematic Review and Meta-Analysis. J Frailty Aging. 2022;11(1):45-50.
99. Granic A, Dismore L, Hurst C, Robinson SM, Sayer AA. Myoprotective Whole Foods, Muscle Health and Sarcopenia: A Systematic Review of Observational and Intervention Studies in Older Adults. Nutrients. 2020;12(8).

100. Feng Z, Lugtenberg M, Franse C, Fang X, Hu S, Jin C, et al. Risk factors and protective factors associated with incident or increase of frailty among community-dwelling older adults: A systematic review of longitudinal studies. PLoS One. 2017;12(6):e0178383.
101. Huo J, Wu L, Lv J, Cao H, Gao Q. Effect of fruit intake on functional constipation: A systematic review and meta-analysis of randomized and crossover studies. Front Nutr. 2022;9:1018502.
102. Lee D, Chiavaroli L, Ayoub-Charette S, Khan TA, Zurbau A, Au-Yeung F, et al. Important Food Sources of Fructose-Containing Sugars and Non-Alcoholic Fatty Liver Disease: A Systematic Review and Meta-Analysis of Controlled Trials. Nutrients. 2022;14(14).
103. Bäcklund R, Drake I, Bergström U, Compagno M, Sonestedt E, Turesson C. Diet and the risk of rheumatoid arthritis - A systematic literature review. Semin Arthritis Rheum. 2023;58:152118.
104. Zhai H, Wang Y, Jiang W. Fruit and Vegetable Intake and the Risk of Chronic Obstructive Pulmonary Disease: A Dose-Response Meta-Analysis of Observational Studies. Biomed Res Int. 2020;2020:3783481.
105. Valera-Gran D, Prieto-Botella D, Hurtado-Pomares M, Baladia E, Petermann-Rocha F, Sánchez-Pérez A, et al. The Impact of Foods, Nutrients, or Dietary Patterns on Telomere Length in Childhood and Adolescence: A Systematic Review. Nutrients. 2022;14(19).
106. He K, Li Y, Guo X, Zhong L, Tang S. Food groups and the likelihood of non-alcoholic fatty liver disease: a systematic review and meta-analysis. Br J Nutr. 2020;124(1):1-13.
107. Takagi H. Which should we eat, fruit or vegetables? The association with abdominal aortic aneurysm. Eur J Prev Cardiol. 2020;27(19):2302-7.
108. Lin BB, Lin ME, Huang RH, Hong YK, Lin BL, He XJ. Dietary and lifestyle factors for primary prevention of nephrolithiasis: a systematic review and meta-analysis. BMC Nephrol. 2020;21(1):267.
109. Galiè S, Canudas S, Muralidharan J, García-Gavilán J, Bulló M, Salas-Salvadó J. Impact of Nutrition on Telomere Health: Systematic Review of Observational Cohort Studies and Randomized Clinical Trials. Adv Nutr. 2020;11(3):576-601.
110. Bajalan Z, Alimoradi Z, Moafi F. Nutrition as a Potential Factor of Primary Dysmenorrhea: A Systematic Review of Observational Studies. Gynecol Obstet Invest. 2019;84(3):209-24.
111. Zhang JW, Xiong JP, Xu WY, Sang XT, Huang HC, Bian J, et al. Fruits and vegetables consumption and the risk of gallstone diasease: A systematic review and meta-analysis. Medicine (Baltimore). 2019;98(28):e16404.
112. Salas-Huetos A, Bulló M, Salas-Salvadó J. Dietary patterns, foods and nutrients in male fertility parameters and fecundability: a systematic review of observational studies. Hum Reprod Update. 2017;23(4):371-89.
113. Rafie N, Golpour Hamedani S, Barak F, Safavi SM, Miraghajani M. Dietary patterns, food groups and telomere length: a systematic review of current studies. Eur J Clin Nutr. 2017;71(2):151-8.
114. Murphy MM, Stettler N, Smith KM, Reiss R. Associations of consumption of fruits and vegetables during pregnancy with infant birth weight or small for gestational age births: a systematic review of the literature. Int J Womens Health. 2014;6:899-912.
115. Schoenaker DA, Soedamah-Muthu SS, Mishra GD. The association between dietary factors and gestational hypertension and pre-eclampsia: a systematic review and meta-analysis of observational studies. BMC Med. 2014;12:157.
116. Alsamarrai A, Das SL, Windsor JA, Petrov MS. Factors that affect risk for pancreatic disease in the general population: a systematic review and meta-analysis of prospective cohort studies. Clin Gastroenterol Hepatol. 2014;12(10):1635-44.e5; quiz e103.
117. Major TJ, Topless RK, Dalbeth N, Merriman TR. Evaluation of the diet wide contribution to serum urate levels: meta-analysis of population based cohorts. BMJ. 2018;363:k3951.
118. Guo X, Yang B, Tan J, Jiang J, Li D. Associations of dietary intakes of anthocyanins and berry fruits with risk of type 2 diabetes mellitus: a systematic review and meta-analysis of prospective cohort studies. Eur J Clin Nutr. 2016;70(12):1360-7.
119. De Amicis R, Mambrini SP, Pellizzari M, Foppiani A, Bertoli S, Battezzati A, et al. Systematic Review on the Potential Effect of Berry Intake in the Cognitive Functions of Healthy People. Nutrients. 2022;14(14).

120. Sweeney M, Burns G, Sturgeon N, Mears K, Stote K, Blanton C. The Effects of Berry Polyphenols on the Gut Microbiota and Blood Pressure: A Systematic Review of Randomized Clinical Trials in Humans. Nutrients. 2022;14(11).
121. Bonyadi N, Dolatkhah N, Salekzamani Y, Hashemian M. Effect of berry-based supplements and foods on cognitive function: a systematic review. Sci Rep. 2022;12(1):3239.
122. Wang Y, Haskell-Ramsay C, Gallegos JL, Lodge JK. Effects of chronic consumption of specific fruit (berries, cherries and citrus) on cognitive health: a systematic review and meta-analysis of randomised controlled trials. Eur J Clin Nutr. 2023;77(1):7-22.
123. Xu L, Tian Z, Chen H, Zhao Y, Yang Y. Anthocyanins, Anthocyanin-Rich Berries, and Cardiovascular Risks: Systematic Review and Meta-Analysis of 44 Randomized Controlled Trials and 15 Prospective Cohort Studies. Front Nutr. 2021;8:747884.
124. Wang Y, Gallegos JL, Haskell-Ramsay C, Lodge JK. Effects of chronic consumption of specific fruit (berries, citrus and cherries) on CVD risk factors: a systematic review and meta-analysis of randomised controlled trials. Eur J Nutr. 2021;60(2):615-39.
125. Martini D, Marino M, Angelino D, Del Bo' C, Del Rio D, Riso P, et al. Role of berries in vascular function: a systematic review of human intervention studies. Nutr Rev. 2020;78(3):189-206.
126. Heneghan C, Kiely M, Lyons J, Lucey A. The Effect of Berry-Based Food Interventions on Markers of Cardiovascular and Metabolic Health: A Systematic Review of Randomized Controlled Trials. Mol Nutr Food Res. 2018;62(1).
127. Luís Â, Domingues F, Pereira L. Association between berries intake and cardiovascular diseases risk factors: a systematic review with meta-analysis and trial sequential analysis of randomized controlled trials. Food Funct. 2018;9(2):740-57.
128. García-Conesa MT, Chambers K, Combet E, Pinto P, Garcia-Aloy M, Andrés-Lacueva C, et al. Meta-Analysis of the Effects of Foods and Derived Products Containing Ellagitannins and Anthocyanins on Cardiometabolic Biomarkers: Analysis of Factors Influencing Variability of the Individual Responses. Int J Mol Sci. 2018;19(3).
129. Huang H, Chen G, Liao D, Zhu Y, Xue X. Effects of Berries Consumption on Cardiovascular Risk Factors: A Meta-analysis with Trial Sequential Analysis of Randomized Controlled Trials. Sci Rep. 2016;6:23625.
130. Darooghegi Mofrad M, Mozaffari H, Askari MR, Amini MR, Jafari A, Surkan PJ, et al. Potato Consumption and Risk of Site-Specific Cancers in Adults: A Systematic Review and Dose-Response Meta-Analysis of Observational Studies. Adv Nutr. 2021;12(5):1705-22.
131. Darooghegi Mofrad M, Milajerdi A, Sheikhi A, Azadbakht L. Potato consumption and risk of all cause, cancer and cardiovascular mortality: a systematic review and dose-response meta-analysis of prospective cohort studies. Crit Rev Food Sci Nutr. 2020;60(7):1063-76.
132. Schwingshackl L, Schwedhelm C, Hoffmann G, Boeing H. Potatoes and risk of chronic disease: a systematic review and dose-response meta-analysis. Eur J Nutr. 2019;58(6):2243-51.
133. Guo F, Zhang Q, Jiang H, He Y, Li M, Ran J, et al. Dietary potato intake and risks of type 2 diabetes and gestational diabetes mellitus. Clin Nutr. 2021;40(6):3754-64.
134. Quan W, Zeng M, Jiao Y, Li Y, Xue C, Liu G, et al. Western Dietary Patterns, Foods, and Risk of Gestational Diabetes Mellitus: A Systematic Review and Meta-Analysis of Prospective Cohort Studies. Adv Nutr. 2021;12(4):1353-64.
135. Quan W, Jiao Y, Xue C, Li Y, Wang Z, Zeng M, et al. Processed potatoes intake and risk of type 2 diabetes: a systematic review and meta-analysis of nine prospective cohort studies. Crit Rev Food Sci Nutr. 2022;62(5):1417-25.
136. Borch D, Juul-Hindsgaul N, Veller M, Astrup A, Jaskolowski J, Raben A. Potatoes and risk of obesity, type 2 diabetes, and cardiovascular disease in apparently healthy adults: a systematic review of clinical intervention and observational studies. Am J Clin Nutr. 2016;104(2):489-98.
137. Akesson A, Andersen LF, Kristjánsdóttir AG, Roos E, Trolle E, Voutilainen E, et al. Health effects associated with foods characteristic of the Nordic diet: a systematic literature review. Food Nutr Res. 2013;57.
138. Fardet A, Richonnet C, Mazur A. Association between consumption of fruit or processed fruit and chronic diseases and their risk factors: a systematic review of meta-analyses. Nutr Rev. 2019;77(6):376-87.

139. Auerbach BJ, Dibey S, Vallila-Buchman P, Kratz M, Krieger J. Review of 100% Fruit Juice and Chronic Health Conditions: Implications for Sugar-Sweetened Beverage Policy. Adv Nutr. 2018;9(2):78-85.
140. Kazemi A, Soltani S, Mokhtari Z, Khan T, Golzarand M, Hosseini E, et al. The relationship between major food sources of fructose and cardiovascular disease, cancer, and all-cause mortality: a systematic review and dose-response meta-analysis of cohort studies. Crit Rev Food Sci Nutr. 2021:1-14.
141. Farvid MS, Barnett JB, Spence ND. Fruit and vegetable consumption and incident breast cancer: a systematic review and meta-analysis of prospective studies. Br J Cancer. 2021;125(2):284-98.
142. Li Y, Guo L, He K, Huang C, Tang S. Consumption of sugar-sweetened beverages and fruit juice and human cancer: a systematic review and dose-response meta-analysis of observational studies. J Cancer. 2021;12(10):3077-88.
143. Pan B, Ge L, Lai H, Wang Q, Zhang Q, Yin M, et al. Association of soft drink and 100% fruit juice consumption with all-cause mortality, cardiovascular diseases mortality, and cancer mortality: A systematic review and dose-response meta-analysis of prospective cohort studies. Crit Rev Food Sci Nutr. 2021:1-12.
144. Llaha F, Gil-Lespinard M, Unal P, de Villasante I, Castañeda J, Zamora-Ros R. Consumption of Sweet Beverages and Cancer Risk. A Systematic Review and Meta-Analysis of Observational Studies. Nutrients. 2021;13(2).
145. Imamura F, O'Connor L, Ye Z, Mursu J, Hayashino Y, Bhupathiraju SN, et al. Consumption of sugar sweetened beverages, artificially sweetened beverages, and fruit juice and incidence of type 2 diabetes: systematic review, meta-analysis, and estimation of population attributable fraction. Br J Sports Med. 2016;50(8):496-504.
146. Xi B, Li S, Liu Z, Tian H, Yin X, Huai P, et al. Intake of fruit juice and incidence of type 2 diabetes: a systematic review and meta-analysis. PLoS One. 2014;9(3):e93471.
147. Frantsve-Hawley J, Bader JD, Welsh JA, Wright JT. A systematic review of the association between consumption of sugar-containing beverages and excess weight gain among children under age 12. J Public Health Dent. 2017;77 Suppl 1:S43-S66.
148. Auerbach BJ, Wolf FM, Hikida A, Vallila-Buchman P, Littman A, Thompson D, et al. Fruit Juice and Change in BMI: A Meta-analysis. Pediatrics. 2017;139(4).
149. Pan A, Malik VS, Hao T, Willett WC, Mozaffarian D, Hu FB. Changes in water and beverage intake and long-term weight changes: results from three prospective cohort studies. Int J Obes (Lond). 2013;37(10):1378-85.
150. Crowe-White K, O'Neil CE, Parrott JS, Benson-Davies S, Droke E, Gutschall M, et al. Impact of 100% Fruit Juice Consumption on Diet and Weight Status of Children: An Evidence-based Review. Crit Rev Food Sci Nutr. 2016;56(5):871-84.
151. Semnani-Azad Z, Khan TA, Blanco Mejia S, de Souza RJ, Leiter LA, Kendall CWC, et al. Association of Major Food Sources of Fructose-Containing Sugars With Incident Metabolic Syndrome: A Systematic Review and Meta-analysis. JAMA Netw Open. 2020;3(7):e209993.
152. Liu Q, Ayoub-Charette S, Khan TA, Au-Yeung F, Blanco Mejia S, de Souza RJ, et al. Important Food Sources of Fructose-Containing Sugars and Incident Hypertension: A Systematic Review and Dose-Response Meta-Analysis of Prospective Cohort Studies. J Am Heart Assoc. 2019;8(24):e010977.
153. Ayoub-Charette S, Liu Q, Khan TA, Au-Yeung F, Blanco Mejia S, de Souza RJ, et al. Important food sources of fructose-containing sugars and incident gout: a systematic review and meta-analysis of prospective cohort studies. BMJ Open. 2019;9(5):e024171.
154. Salas MM, Nascimento GG, Vargas-Ferreira F, Tarquinio SB, Huysmans MC, Demarco FF. Diet influenced tooth erosion prevalence in children and adolescents: Results of a meta-analysis and meta-regression. J Dent. 2015;43(8):865-75.
155. D'Elia L, Dinu M, Sofi F, Volpe M, Strazzullo P, SINU Working Group EdbS. 100% Fruit juice intake and cardiovascular risk: a systematic review and meta-analysis of prospective and randomised controlled studies. Eur J Nutr. 2021;60(5):2449-67.
156. Liska D, Kelley M, Mah E. 100% Fruit Juice and Dental Health: A Systematic Review of the Literature. Front Public Health. 2019;7:190.
157. Blanch N, Clifton PM, Keogh JB. A systematic review of vascular and endothelial function: effects of fruit, vegetable and potassium intake. Nutr Metab Cardiovasc Dis. 2015;25(3):253-66.

158. Ayoub-Charette S, Chiavaroli L, Liu Q, Khan TA, Zurbau A, Au-Yeung F, et al. Different Food Sources of Fructose-Containing Sugars and Fasting Blood Uric Acid Levels: A Systematic Review and Meta-Analysis of Controlled Feeding Trials. J Nutr. 2021;151(8):2409-21.
159. Cowan SF, Leeming ER, Sinclair A, Dordevic AL, Truby H, Gibson SJ. Effect of whole foods and dietary patterns on markers of subclinical inflammation in weight-stable overweight and obese adults: a systematic review. Nutr Rev. 2020;78(1):19-38.
160. Choo VL, Viguiliouk E, Blanco Mejia S, Cozma AI, Khan TA, Ha V, et al. Food sources of fructose-containing sugars and glycaemic control: systematic review and meta-analysis of controlled intervention studies. BMJ. 2018;363:k4644.
161. Murphy MM, Barrett EC, Bresnahan KA, Barraj LM. 100 % Fruit juice and measures of glucose control and insulin sensitivity: a systematic review and meta-analysis of randomised controlled trials. J Nutr Sci. 2017;6:e59.
162. Tonin FS, Steimbach LM, Wiens A, Perlin CM, Pontarolo R. Impact of Natural Juice Consumption on Plasma Antioxidant Status: A Systematic Review and Meta-Analysis. Molecules. 2015;20(12):22146-56.
163. Wang B, Liu K, Mi M, Wang J. Effect of fruit juice on glucose control and insulin sensitivity in adults: a meta-analysis of 12 randomized controlled trials. PLoS One. 2014;9(4):e95323.
164. Crowe-White K, Parrott JS, Stote KS, Gutschall M, Benson-Davies S, Droke E, et al. Metabolic impact of 100% fruit juice consumption on antioxidant/oxidant status and lipid profiles of adults: An Evidence-Based review. Crit Rev Food Sci Nutr. 2017;57(1):152-62.
165. Liu K, Xing A, Chen K, Wang B, Zhou R, Chen S, et al. Effect of fruit juice on cholesterol and blood pressure in adults: a meta-analysis of 19 randomized controlled trials. PLoS One. 2013;8(4):e61420.
166. Zheng J, Zhou Y, Li S, Zhang P, Zhou T, Xu DP, et al. Effects and Mechanisms of Fruit and Vegetable Juices on Cardiovascular Diseases. Int J Mol Sci. 2017;18(3).
